# Characterization of RyDEN (C19orf66) as an Interferon-Stimulated Cellular Inhibitor against Dengue Virus Replication

**DOI:** 10.1371/journal.ppat.1005357

**Published:** 2016-01-06

**Authors:** Youichi Suzuki, Wei-Xin Chin, Qi'En Han, Koji Ichiyama, Ching Hua Lee, Zhi Wen Eyo, Hirotaka Ebina, Hirotaka Takahashi, Chikako Takahashi, Beng Hui Tan, Takayuki Hishiki, Kenji Ohba, Toshifumi Matsuyama, Yoshio Koyanagi, Yee-Joo Tan, Tatsuya Sawasaki, Justin Jang Hann Chu, Subhash G. Vasudevan, Kouichi Sano, Naoki Yamamoto

**Affiliations:** 1 Department of Microbiology, Yong Loo Lin School of Medicine, National University of Singapore, Singapore; 2 Emerging Infectious Disease Program, Duke-NUS Graduate Medical School, Singapore; 3 Laboratory of Viral Pathogenesis, Institute for Virus Research, Kyoto University, Kyoto, Japan; 4 Proteo-Science Center, Ehime University, Matsuyama, Japan; 5 Laboratory of Primate Model, Experimental Research Center for Infectious Diseases, Institute for Virus Research, Kyoto University, Kyoto, Japan; 6 Department of Microbiology and Infection Control, Osaka Medical College, Takatsuki, Japan; The Rockefeller University, UNITED STATES

## Abstract

Dengue virus (DENV) is one of the most important arthropod-borne pathogens that cause life-threatening diseases in humans. However, no vaccine or specific antiviral is available for dengue. As seen in other RNA viruses, the innate immune system plays a key role in controlling DENV infection and disease outcome. Although the interferon (IFN) response, which is central to host protective immunity, has been reported to limit DENV replication, the molecular details of how DENV infection is modulated by IFN treatment are elusive. In this study, by employing a gain-of-function screen using a type I IFN-treated cell-derived cDNA library, we identified a previously uncharacterized gene, *C19orf66*, as an IFN-stimulated gene (ISG) that inhibits DENV replication, which we named Repressor of yield of DENV (RyDEN). Overexpression and gene knockdown experiments revealed that expression of RyDEN confers resistance to all serotypes of DENV in human cells. RyDEN expression also limited the replication of hepatitis C virus, Kunjin virus, Chikungunya virus, herpes simplex virus type 1, and human adenovirus. Importantly, RyDEN was considered to be a crucial effector molecule in the IFN-mediated anti-DENV response. When affinity purification-mass spectrometry analysis was performed, RyDEN was revealed to form a complex with cellular mRNA-binding proteins, poly(A)-binding protein cytoplasmic 1 (PABPC1), and La motif-related protein 1 (LARP1). Interestingly, PABPC1 and LARP1 were found to be positive modulators of DENV replication. Since RyDEN influenced intracellular events on DENV replication and, suppression of protein synthesis from DENV-based reporter construct RNA was also observed in RyDEN-expressing cells, our data suggest that RyDEN is likely to interfere with the translation of DENV via interaction with viral RNA and cellular mRNA-binding proteins, resulting in the inhibition of virus replication in infected cells.

## Author Summary

Dengue is the most common arthropod-borne viral infection and is spreading to new areas every year. Its causative agent, dengue virus (DENV), has immunologically distinct serotypes that increase the risk of life-threatening diseases such as dengue hemorrhagic fever. However, an effective medication for dengue has not yet been established. There is, therefore, an urgent need to develop new antivirals and vaccines against DENV. Here, we have characterized C19orf66, named Repressor of yield of DENV (RyDEN), as a cellular gene that inhibits the replication of all DENV serotypes. The expression of RyDEN was found to be upregulated by interferon (IFN) treatment and played a critical role in the IFN-mediated anti-DENV response. We also found that RyDEN was likely to block the protein translation of DENV RNA through its association with cellular mRNA-binding proteins and viral RNA. Intriguingly, replication of several other viruses, such as hepatitis C virus, Kunjin virus, and Chikungunya virus, was also limited by RyDEN expression. Thus, this study describes a novel mechanism of an IFN-inducible inhibitory factor for DENV and provides the basis for future development of broad-spectrum antivirals against infectious viral diseases, including dengue.

## Introduction

Dengue virus (DENV) is a mosquito-borne virus belonging to the genus *Flavivirus*, which is a large family of enveloped, positive-stranded RNA viruses. DENV has four antigenically distinct serotypes (DENV-1 to -4); all serotypes are able to cause dengue fever (DF) and dengue hemorrhagic fever (DHF) in humans. While primary infection with one of the four DENV serotypes is often asymptomatic or causes self-limiting DF, due to the presence of non- or sub-neutralizing antibodies produced during the primary infection, a secondary infection with a different serotype increases the risk of a more severe form of dengue infection, such as life-threatening DHF and dengue shock syndrome (DSS). However, there is currently no effective vaccine or specific antiviral treatment available for dengue prevention and control [1].

At the cellular level, DENV infection begins with entry via receptor-mediated endocytosis, followed by particle disassembly to release an ~11-kb single-stranded RNA genome into the cytoplasm. The viral genomic RNA contains an open reading frame (ORF) encoding a single polyprotein, which is flanked by a capped 5’ untranslated region (UTR) and a non-polyadenylated 3’UTR, and serves as a template for the translation of a viral precursor protein. The single polypeptide is then cleaved co- and post-translationally into three structural (C, prM, and E) and seven non-structural (NS) proteins (NS1, NS2A, NS2B, NS3, NS4A, NS4B, and NS5). The structural proteins are used for the assembly of virus particles, while the NS proteins are mainly involved in synthesis of the viral RNA genome and the further translation process during DENV infection [2].

Many host factors have been reportedly implicated in the replication of DENV; however, the biological relevance of those factors in *in vivo* infection and the pathogenesis of DENV has not been fully addressed [3,4]. Meanwhile, it has also become apparent that host cells may harbor factors whose expressions potentially restrict DENV replication. In this regard, the induction of the interferon (IFN) response is considered to be the first line of defense against an invading DENV [5]. DENV infection is able to induce the IFN response, probably through the recognition of viral genomic RNA by intracellular receptors such as TLR-3, RIG-I, and MDA-5 [6–8], which in turn triggers a cellular antiviral state that suppresses the early replication and subsequent spread of DENV. Several *in vitro* studies have reported that the establishment of a DENV infection is capable of antagonizing IFN signaling cascades by employing viral NS proteins [9–14]. However, pretreatment of human cells with type I (IFN-α and IFN-β) or type II (IFN-γ) IFNs has been shown to limit the replication of DENV [15]. Also, mice deficient in IFN receptors [16] or an IFN signaling component, signal transducer, and activator of transcription 2 (STAT2) [17,18] are reported to be highly susceptible to DENV infection. Given additional evidence that DHF/DSS patients have higher levels of circulating IFN-α and IFN-γ as compared to DF patients [19–21], IFN response is likely to play a key role in controlling DENV replication *in vivo* [22].

The antiviral effect of IFN is known to be mediated by interferon-stimulated genes (ISGs), which disrupt various steps of virus replication [23]. So far, hundreds of genes have been classified as ISGs; among them, a number of ISGs have been demonstrated to restrict divergent families of viruses, including flaviviruses [23–26]. As for DENV, gene overexpression and knockdown studies have reported that several human ISGs, including interferon-inducible transmembrane proteins (IFITMs), ISG15, ISG20, Viperin, and BST2, have suppressive effects against *in vitro* virus infection [27–32]. Additionally, a recent large-scale screening study using a library of ISG comprising more than 350 genes revealed that at least 10 ISGs were potent cellular inhibitors of DENV replication that modulate DENV infection in the early or late stage of virus replication [33]. Although the precise mechanisms of action of the anti-DENV ISGs are not yet clear, many of them are likely to function as effector molecules that directly interfere with viral components during infection [23]. Therefore, we believe that understanding how IFN-inducible effector molecules restrict virus infection will be the molecular basis for developing new antiviral agents and vaccines against DENV.

This study aimed to identify new cellular suppressive factors against DENV infection by a gain-of-function screen using a cDNA library derived from type I IFN-treated human cells. We then found that a previously uncharacterized cellular gene, C19orf66, named RyDEN (Repressor of yield of DENV), conferred resistance to all serotypes of DENV in human cells. RyDEN was considered to be an ISG whose expression was essential for the full activity of the type I IFN-mediated suppression of DENV replication. Other than its impact on DENV, overexpression of RyDEN in human cells limited the replication of several RNA and DNA viruses. Interestingly, RyDEN was found to form a complex with cellular mRNA binding proteins, PABPC1 and LARP1, which are required for the efficient replication of DENV. Moreover, RyDEN was likely to interact with DENV RNA and impair the protein translation of viral RNA. Our data demonstrate a novel mechanism of ISG in the inhibition of DENV infection.

## Results

### Isolation of anti-DENV factors by a gain-of-function cDNA screen

**Fig 1:**
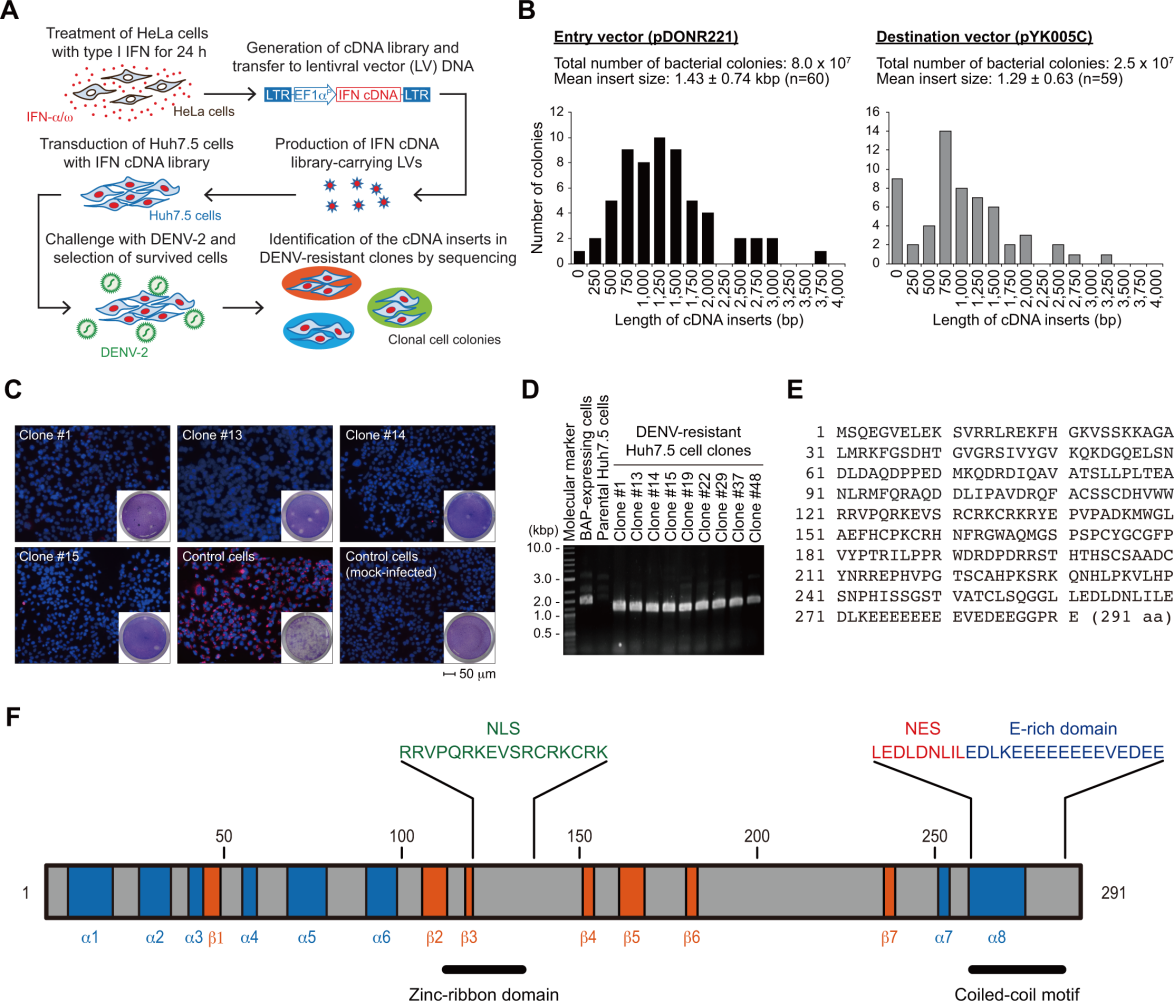
Identification of RyDEN. (A) Procedure for gain-of-function screen. The cDNA library was generated from mRNA of IFN-α/ω-treated HeLa cells and transferred into a lentiviral vector by the Gateway recombination system. Infectious lentiviral vectors carrying the IFN cDNA library were produced as a VSV-G-pseudotyped virus and used to transduce DENV-susceptible Huh7.5 cells. cDNA library-expressing Huh7.5 cells were then challenged with DENV-2 at an MOI of 1, and cell colonies that survived DENV-induced cell death were collected. (B) Histogram analysis of cDNA fragments in library vectors. The entry vector (pDONR221, left panel) and destination vector (pYK005C, right panel) recombinated with the Gateway-compatible cDNA library were applied to *Escherichia coli* (*E*. *coli)*, and the cells were spread onto LB plates to develop bacterial colonies. The cDNA fragments in individual colonies were amplified by PCR using primers described in Materials and Methods and visualized with agarose gel electrophoresis. The size of the PCR fragment was estimated by comparing the migration distance of the DNA molecular weight markers. Up to sixty colonies were picked up from each vector-transformed *E*. *coli* plate and analyzed. (C) Validation of DENV-resistant cell clones. Surviving clones obtained from (A) were seeded in a chamber slide and infected with DENV-2 at an MOI of 5. Two days after infection, cells were fixed with paraformaldehyde, permeabilized, and stained with anti-dsRNA antibody, followed by detection with Alexa Fluor 488-conjugated secondary antibody (red). Cell nuclei were stained with DAPI (blue). Representative merged images using four surviving clones (#1, 13, 14, and 15) and control cells (bacterial alkaline phosphatase [BAP]-expressing Huh7.5 cells) are shown. In a parallel experiment, the culture supernatant of infected cells was harvested 2 days after infection and subjected to plaque assay to measure the virus titer (insets). (D) Amplification of cDNA from DENV-resistant cells. Genomic DNA was isolated from cell clones, whose resistant property had been confirmed in (B), and cDNA was amplified by PCR using primers specific to the lentiviral vector. PCR products were separated by agarose gel electrophoresis and visualized by ethidium bromide staining. (E) Amino acid sequence of RyDEN. (F) Predicted domain organization of RyDEN. RyDEN protein (291 amino acid) was suggested to contain eight α-helixes (blue), seven β-strands (orange), NLS (121–137), NES (261–269), zinc-ribbon domain (112–135), and coiled-coil motif (261–285). A unique glutamic acid-rich (E-rich) domain was also found in the C-terminus.

**Fig 2:**
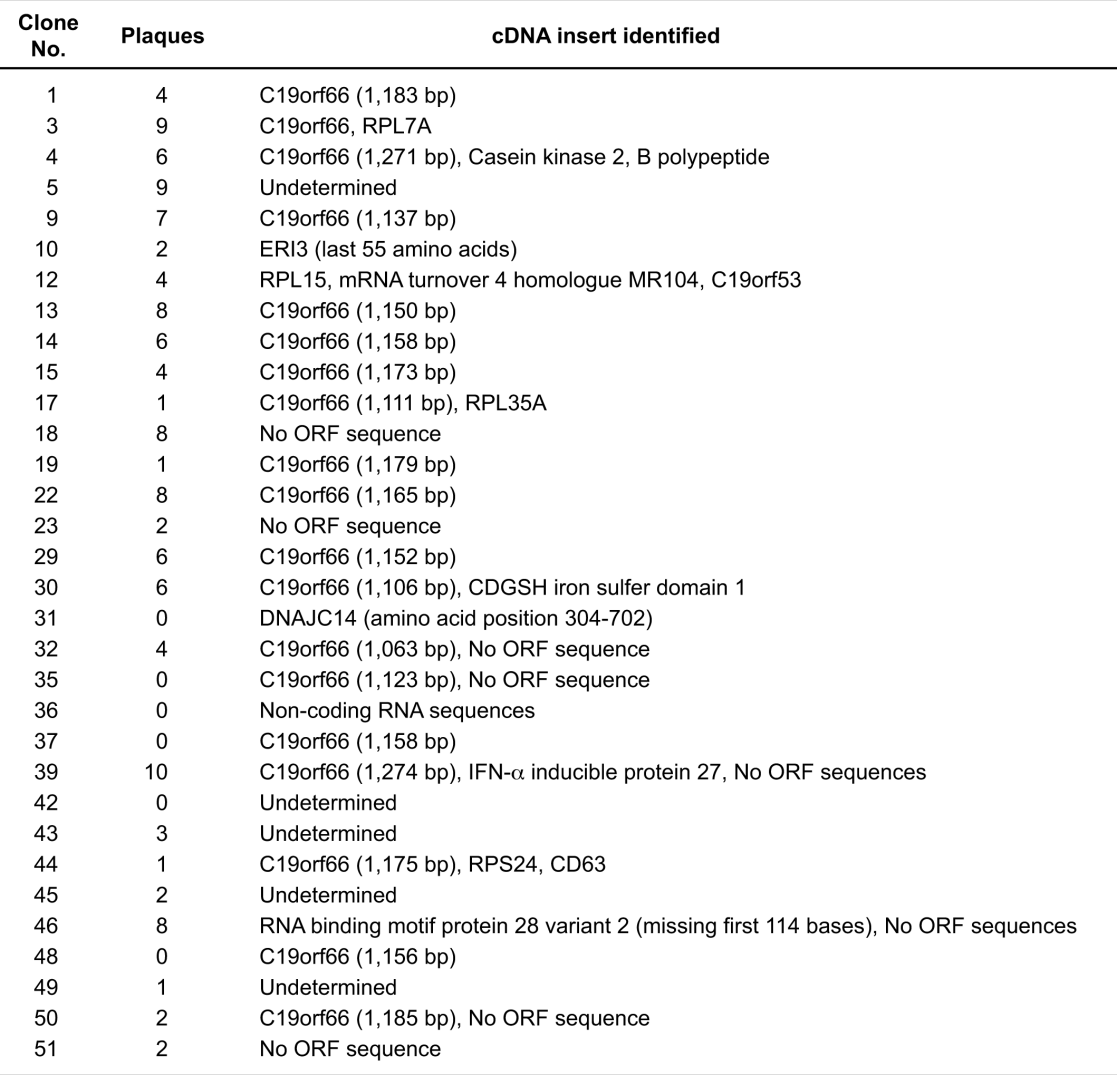
Summary of cDNAs recovered from DENV-resistant Huh7.5 cell clones. Fifty-two colonies of clonal Huh7.5 cells that survived a chellenge infection of DENV-2 were infected with DENV at an MOI of 1, and culture supernatants of infected cells were subjected to plaque assay. mRNA were isolated from cells whose supernatant formed 10 or less DENV plaques less (DENV-resistant clones, total 32 clones), and cDNA inserts expressing in the clones were analyzed by sequencing. More than 50 plaques were formed with a supernatant of control protein (BAP)-expressing Huh7.5 cells.

It has been demonstrated that pretreatment with type I IFN protects human cells from DENV infection *in vitro* [15,34]. In order to identify anti-DENV effector molecule(s) in the IFN response, a pool of cDNA was generated from the mRNA of HeLa cells that had been treated with type I IFN (a mixture of human IFNα and ω [Sigma]) and transferred to a lentiviral vector, pYK005C [35], by the Gateway recombination system (Fig 1A). The mean sizes of the IFN-derived cDNA in the Gateway entry (i.e., pDONR22) and destination (pYK005C) vectors were 1.43±0.74 and 1.29±0.63 kbp, respectively (Fig 1B). Infectious lentiviral vectors carrying the cDNA library were produced as a vesicular stomatitis virus G protein (VSV-G) pseudotype and used to transduce a human hepatoma cell line, Huh7.5, which exhibited a massive cytopathic effect with DENV-2 infection (S1 Fig). Transduced cells were then challenged with DENV-2 (Singapore isolate EDEN2 3295 [36]) at a multiplicity of infection (MOI) of 1, and surviving cells were selected (Fig 1A). From the initial screen, a total of 52 surviving cell clones were collected and further verified for their resistance against DENV infection. Plaque assay revealed that, among the 52 cell clones, inhibition of DENV-2 replication was still observed in 32 clones (Fig 2). Reduced replication of DENV in the clones was also confirmed by immunofluorescent analysis (IFA) using anti-double-stranded (ds) RNA antibodies (Fig 1C). PCR amplification and subsequent sequencing analysis using a BLAST search revealed that cDNA from 19 of 32 DENV-resistant clones (59.3%) contained an ORF of a previously uncharacterized gene on chromosome 19, *C19orf66*, in the integrated pYK005C vector genome (Fig 1D and 1E). Because the inhibitory effect of C19orf66 on DENV replication was confirmed by the following experiments, we named this gene Repressor of yield of DENV (RyDEN).

### Resistance against DENV in human cells conferred by expression of the RyDEN

RyDEN/C19orf66 is an eight-exon gene located on genomic region 19p13.2. This gene spans approximately 7.1 kb in the human genome and encodes a 291 amino acid protein in its ORF (Fig 1E). However, the functional characteristic of the protein product of the RyDEN gene is unknown. A secondary structure prediction by the JPred program (http://www.compbio.dundee.ac.uk/jpred/) represented RyDEN as consisting of eight α-helixes and seven β-strands (Fig 1F). The RyDEN amino acid sequence was also predicted to contain a nuclear localization signal (NLS, 121–137, by cNLS Mapper [http://nls-mapper.iab.keio.ac.jp/cgi-bin/NLS_Mapper_form.cgi]), a nuclear export signal (NES, 261–269, by NetNES [http://www.cbs.dtu.dk/services/NetNES/]), a zinc-ribbon domain (112–135) that is defined by CXXC(H)-15/17-CXXC [37] and a coiled-coil motif (261–286) (Fig 1F). In addition, a characteristic glutamic acid (E)-rich domain was found in the C-terminal region (274–286, Fig 1F).

**Fig 3:**
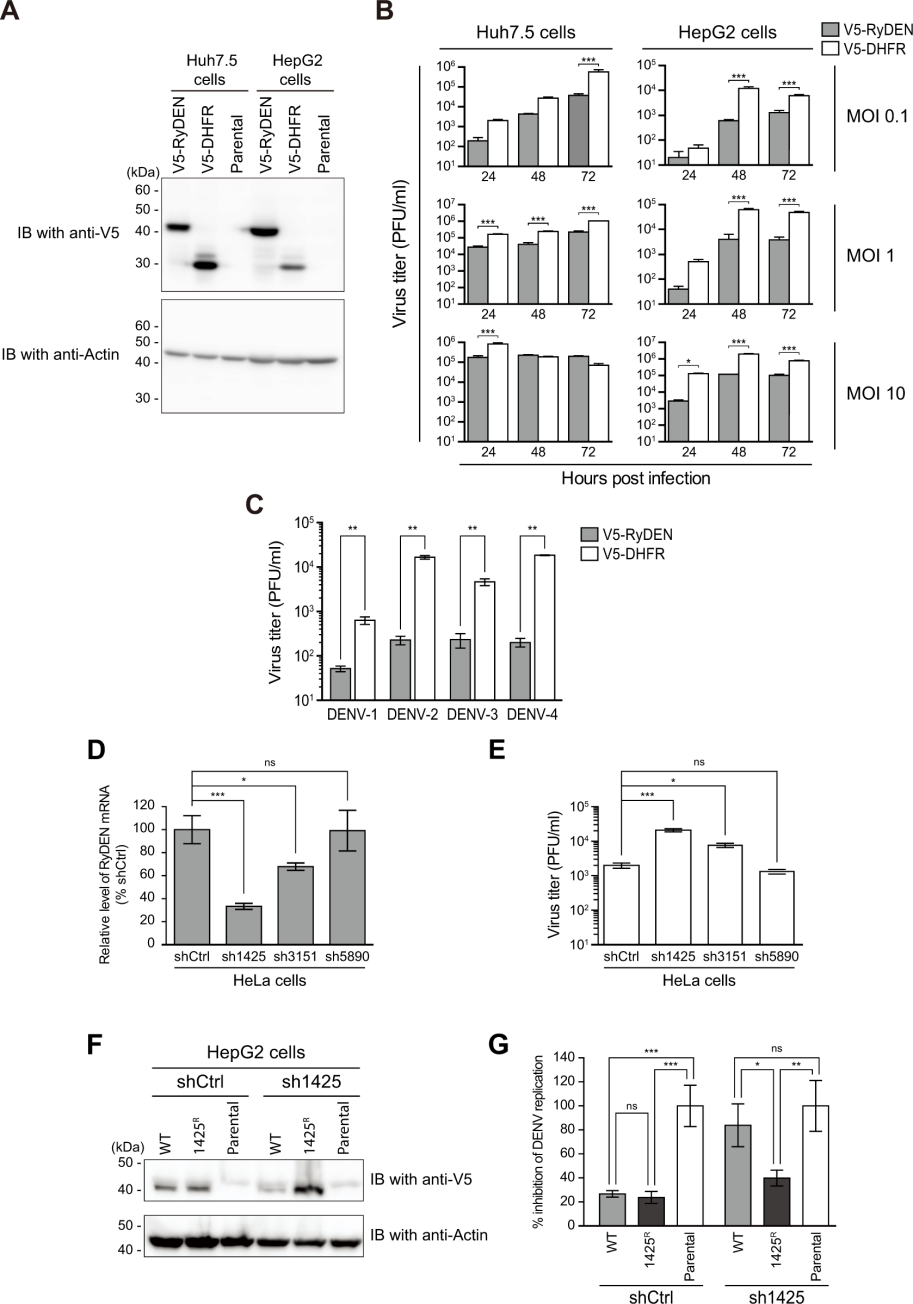
Inhibition of DENV replication by RyDEN expression. (A) Establishment of human cell lines stably expressing V5-tagged RyDEN. Huh7.5 and HepG2 cells expressing V5-RyDEN or V5-DHFR were created by lentiviral vector transduction and blasticidin selection. Expression of V5-tagged proteins was analyzed by immunoblotting (IB). Masses of molecular weight standards are indicated at left. (B) Replication of DENV-2 in stable cell lines. Huh7.5 (left panels) and HepG2 (right panels) cells expressing V5-RyDEN (gray) or V5-DHFR (white) were infected with DENV-2 (Singapore isolate) at MOIs of 0.1 (top), 1 (middle), and 10 (bottom), and virus replication was monitored until 72 h after infection. Infectious titers in culture supernatants were quantified by plaque assay. Note that, at an MOI of 10, the virus titer for V5-DHFR-expressing Huh7.5 cells peaked at the first day, and by the second day, a large proportion of the cells exhibited massive CPE, whereas V5-RyDEN-expressing Huh7.5 cells that displayed resistance to DENV-induced CPE produced steady level of viruses even after 2 days. (C) Inhibitory effect of RyDEN against all DENV serotypes. HepG2 cells expressing V5-RyDEN (gray bars) and V5-DHFR (white bars) were infected with DENV-1, -3, -4 (Singapore isolates), or -2 (New Guinea strain [NGC]) at an MOI of 0.1, and the virus titer was determined 2 days after infection. (D) shRNA-based knockdown of RyDEN mRNA. HeLa cells were transduced with lentiviral vectors expressing three different shRNA sequences against RyDEN mRNA (sh1425, sh3151, or sh5890) and subjected to puromycin selection to create stable cell lines. The expression level of RyDEN mRNA in RyDEN shRNA and non-targeting control shRNA (shCtrl)-expressing cells were analyzed by qRT-PCR analysis and normalized with GAPDH mRNA levels. (E) Replication efficiency of DENV in the knockdown cells. shRNA-expressing HeLa cells were infected with DENV-2 at an MOI of 1, and the viral titer in culture supernatant 2 days after infection was quantified by plaque assay. (F) Add-back of shRNA-resistant RyDEN in knockdown cells. Control shRNA (shCtrl, left lanes) and RyDEN shRNA (sh1425, right lanes)-expressing cells were established again using HepG2 cells. Cells were further transduced with lentiviral vectors expressing sh1425-susceptible wild-type (WT) or sh1425-resistant (1425^R^) V5-RyDEN and selected with blasticidin. The expression of V5-tagged RyDEN was analyzed by immunoblotting (IB) analysis. Masses of molecular weight standards are indicated at left. Parental stands for the untransduced shRNA cell line. (G) Effect of the shRNA-resistant RyDEN expression on DENV replication in knockdown cells. Cell lines created in (F) were infected with DENV-2 at an MOI of 0.1, and the virus titer 2 days after infection was determined. The level of virus titer in the culture supernatants of WT and 1425^R^ cells relative to the parent cells (derived from each shRNA-expressing cells) is shown. Statistical significance was determined by two-way ANOVA (B), Student’s *t* test (C), or one-way ANOVA with Dunnett’s multiple comparison test (D, E, and G). ns, no significance (i.e., *P*>0.05).

In order to verify the inhibitory action of RyDEN against DENV, the ORF of RyDEN was cloned back into a lentiviral vector as an N-terminal V5-tagged gene and used to create human cell lines (Huh7.5 and HepG2) that stably expressed V5-RyDEN (Fig 3A). When the cell lines were infected with three doses of DENV-2 (MOIs of 0.1, 1, and 10), virus replication was significantly suppressed, reducing the virus titer by ~43-fold, as compared to the control protein (V5-tagged bacterial dihydrofolate reductase [DHFR])-expressing cells (Fig 3B). Although a more potent inhibitory effect of V5-RyDEN expression was observed in HepG2 cells than in Huh7.5 cells (Fig 3B), it was presumed that this difference occurred due to a higher susceptibility of Huh7.5 cells to DENV infection or a higher expression of V5-RyDEN in HepG2 cells (Fig 3A). DENV inhibition by RyDEN expression was also observed in HEK293T cells (S2 Fig). The ability of RyDEN to inhibit three other serotypes of DENV (Singapore isolates [36]) and another strain of DENV-2 (New Guinea C [NGC]) was also examined. Results showed that the replication of DENV-1, -2, -3, and -4 were inhibited 12.3-, 72.7-, 20.0-, and 92.3-fold, respectively (Fig 3C).

Using an RNA interference experiment, we next investigated whether endogenous expression of RyDEN acts as a suppressor against DENV. To create RyDEN knockdown cells, lentiviral vectors expressing three different small hairpin RNA (shRNA) sequences against RyDEN mRNA (sh1425, sh3151, and sh5890) or a non-targeting control shRNA (shCtrl) were constructed and used to transduce HeLa cells. Quantitative reverse transcription-PCR (qRT-PCR) analysis showed that the expression levels of endogenous RyDEN mRNA in shRNA1425-, sh3151-, and sh5890-expressing cells were 33.3, 67.8, and 99.2%, respectively, as compared with those of shCtrl-expressing cells (Fig 3D). Following infection of the knockdown cell lines with DENV-2 at an MOI of 1 revealed that virus replication was significantly stimulated by RyDEN silencing, which was in accordance with the depletion efficiency of RyDEN mRNA in the three shRNA cell lines (Fig 3E). This enhancement of DENV replication by the knockdown of endogenous RyDEN was also observed in HepG2 and Huh7.5 cells (S3 Fig). In order to test the specificity and reproducibility of the shRNA experiment, we also created sh1425- and shCtrl-expressing cell lines using HepG2 cells. Then, sh1425-susceptible wild-type (WT) or sh1425-resistant mutant (1425^R^) V5-RyDEN was expressed in the cell lines by the transduction of the lentiviral vector system (Fig 3F). The created cells were challenged with DENV-2. When compared to untransduced (parental) shRNA cells, both V5-RyDEN (i.e., WT and 1425^R^) suppressed DENV replication at a similar levels in shCtrl cells, whereas, in sh1425 cells, a significant reduction of virus replication was observed only with 1425^R^ RyDEN expression but not with WT RyDEN expression (Fig 3G). Taken together, these data conclude that the expression of RyDEN confers resistance to DENV infection in human cells.

### RyDEN is an ISG critical for IFN-mediated anti-DENV response

**Fig 4:**
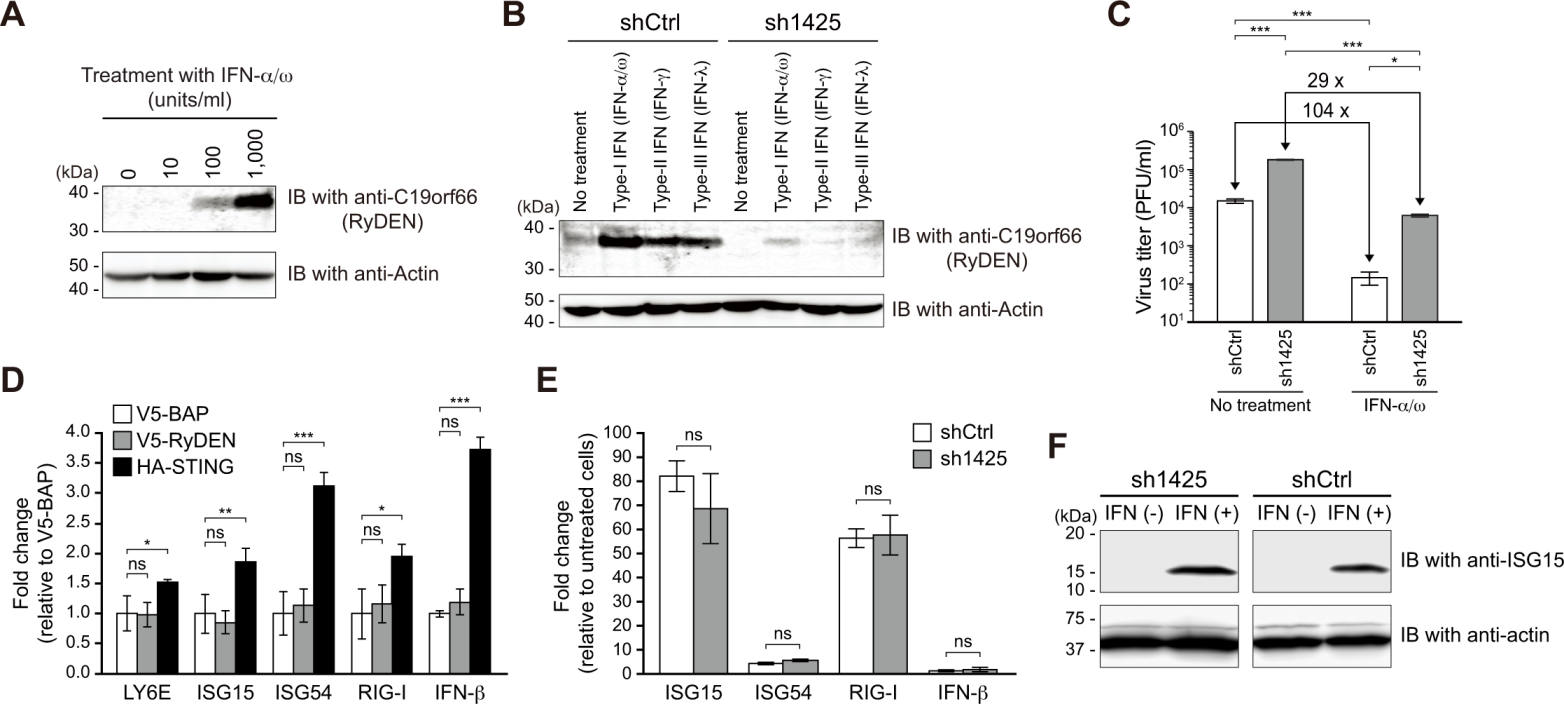
Critical contribution of RyDEN in IFN-mediated anti-DENV response. (A and B) Induction of RyDEN expression by IFN treatments. HeLa (A) and shRNA-expressing HepG2 (B) cells were treated with increasing concentrations (10, 100, and 1,000 units/ml) of IFN-α/ω or a fixed concentration (300 units/ml) of IFN-α/ω, IFN-γ, and IFN-λ1. Cell lysates 24 h after treatment were subjected to immunoblotting (IB) analysis. Masses of molecular weight standards are indicated at left. (C) Effect of RyDEN knockdown on IFN-mediated DENV inhibition. sh1425 (RyDEN knockdown) or shCtrl-expressing HepG2 cells were pretreated with 300 units/ml of IFN-α/ω and, 24 h after infection, exposed to DENV-2 at an MOI of 1. The virus titer of the culture supernatant was measured by plaque assay 2 days after infection. Statistical significance was determined using two-way ANOVA. (D) HepG2 cells were transfected with plasmid DNA-expressing V5-RyDEN (gray bars), V5-BAP (white bars), or HA-STING (black bars). Total RNA was isolated 48 h after transfection and subjected to qRT-PCR analysis for the detection of LY6E, ISG15, ISG54, RIG-I, and IFN-β mRNA. Statistical significance was determined by one-way ANOVA with Dunnett’s multiple comparison test. ns, no significance. (E) RyDEN knockdown (sh1425-expressing, gray bars) and control (shCtrl-expressing, white bars) HeLa cells were cultured in the presence or absence of 1,000 units/ml IFN-α/ω. Total RNA was isolated 24 h after treatment and subjected to qRT-PCR analysis. The levels of gene expression were expressed as the fold change compared to untreated cells. ns, no significance. (F) sh1425- (left panels) and shCtrl-expressing (right panels) HeLa cells treated with IFN-α/ω were subjected to immunoblotting analysis using anti-ISG15 antibodies (top panels). The same blot was also probed with anti-actin antibodies (bottom panels). Masses of molecular weight standards are indicated at left.

Since RyDEN was first identified by a gain-of-function screen using a type I IFN-treated HeLa cell-derived cDNA library (Fig 1), we examined whether RyDEN was upregulated by IFN treatment in human cells. Immunoblotting analysis using a commercially available anti-RyDEN antibodies (anti-C19orf66 rabbit IgG purchased from Abcam) revealed that RyDEN expression was indeed enhanced in HeLa cells in response to the increasing concentration of IFN-α/ω (Fig 4A). In addition to the type I IFN treatment, the expression of RyDEN was also upregulated by treatment with type II (IFN-γ) and type III (IFN-λ) IFNs (Fig 4B). It is notable that the specificity of the anti-RyDEN antibody on treatment with IFNs was confirmed by knockdown of RyDEN mRNA in sh1425-expressing HepG2 cells (Fig 4B). Quantitative analysis of RyDEN mRNA by qRT-PCR showed that upregulation of RyDEN expression by type I IFN treatment was also observed in all of the human cell lines tested; however, the induction level varied among cells (S4 Fig).

Next, we evaluated how RyDEN expression could contribute to IFN-mediated anti-DENV functions using the RyDEN knockdown cell line. As was observed in a previous report [15], pretreatment with IFN-α/ω suppressed the replication of DENV-2 104-fold in control shRNA (shCtrl)-expressing HepG2 cells (Fig 4C, white bars). However, in HeLa cells in which endogenous RyDEN had been depleted by sh1425, type I IFN treatment inhibited DENV infection by only 29% (Fig 4C, gray bars). Thus, these results indicate that RyDEN is an ISG that plays a critical role in the IFN-mediated anti-DENV response in human cells.

In order to test the possibility that RyDEN may be a key component in the IFN signaling pathway, we compared gene expressions of a variety of ISGs between RyDEN and control protein-expressing cells. HepG2 cells were transfected with V5-tagged RyDEN or control BAP -expressing plasmid DNA, and 48 h after transfection, the level of mRNA expression of the ISGs (LY6E, ISG15, ISG54, and RIG-I) and IFN-β were measured by qRT-PCR analysis. The results showed that no significant activation in the mRNA expression of these genes was observed with the V5-RyDEN transfection, whereas the transfection of a stimulator of the interferon gene (STING), an endoplasmic reticulum-associated adaptor molecule regulating the IFN production [38], upregulated the ISGs and the IFN-β gene (Fig 4D). Additionally, a parallel experiment using RyDEN knockdown (sh1425) and control (shCtrl) HeLa cells revealed that gene expressions of ISG15, ISG54, RIG-I, and IFN-β upon treatment with IFN-α/ω were not reduced by the depletion of endogenous RyDEN (Fig 4E).

A recent study reported a unique regulation of ISG expression, in which some host RNA-binding proteins activated the translational process of ISG mRNA [39]. Hence, we also tested whether RyDEN was involved in the translational regulation of ISGs. An immunoblotting analysis against ISG15, which has been reported to restrict DENV replication [30], showed that a comparable level of ISG15 protein expression following type I IFN treatment was detected in RyDEN knockdown (sh1425-expressing) and control (shCtrl-expressing) HeLa cells (Fig 4F). In summary, these results indicate that RyDEN is not a regulator of the IFN response.

### RyDEN’s inhibitory mode of action

**Fig 5:**
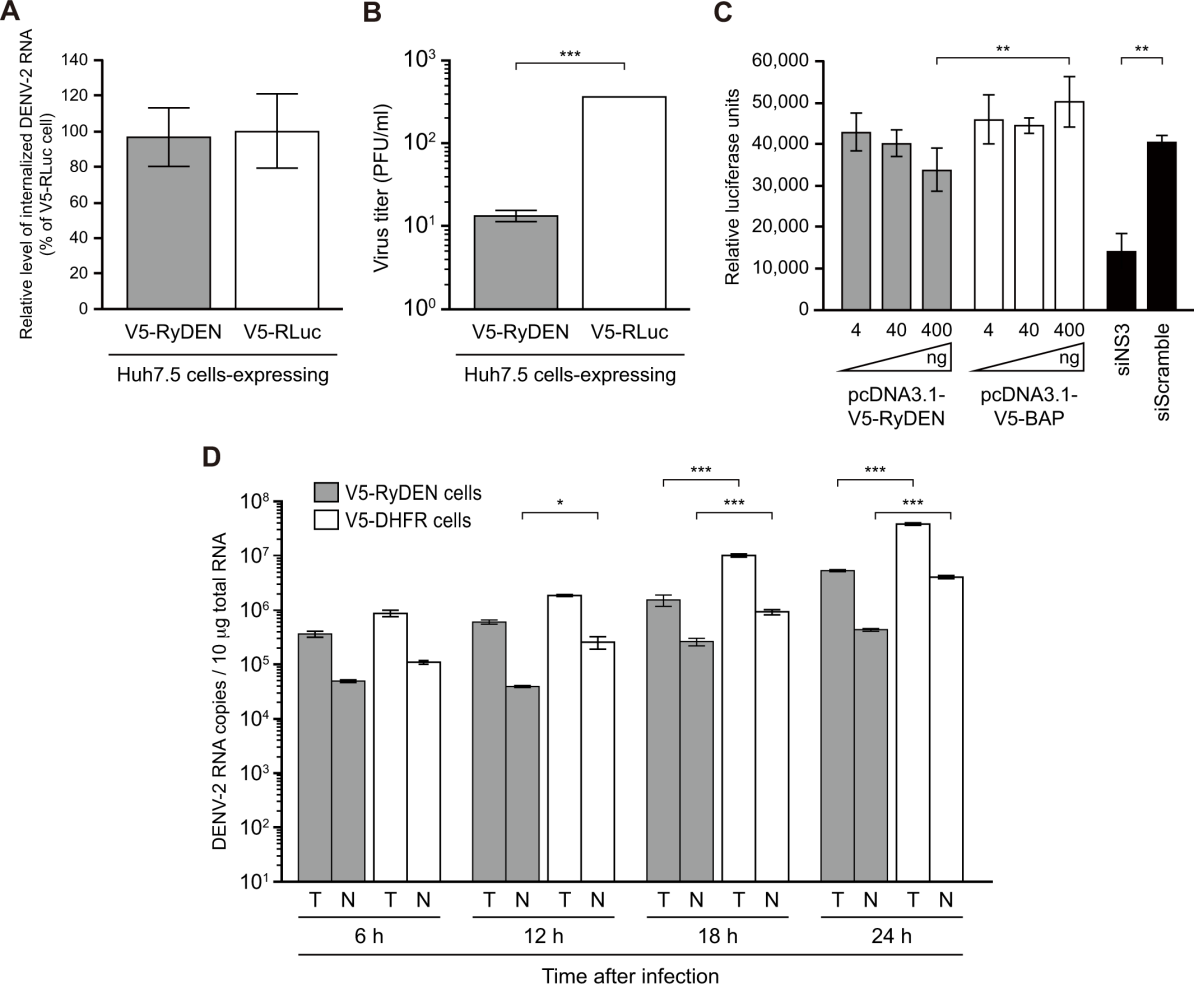
Suppression of intracellular events of DENV infection by RyDEN. (A) Entry assay. V5-RyDEN (gray bar) and V5-RLuc (white bar)-expressing Huh7.5 cells were incubated with DENV-2 at an MOI of 5 at 37°C for 2 h. After washing with ice-cold PBS and a high salt content alkaline solution, total RNA was isolated and subjected to qRT-PCR analysis. The DENV-2 RNA level was normalized with GAPDH mRNA levels. (B) Viral RNA transfection assay. DENV-2 RNA purified from the culture supernatant of infected cells was transfected into V5-RyDEN (gray bar) and V5-RLuc (white bar)-expressing Huh7.5 cells by lipofection. Three days after transection, the culture supernatant was collected and subjected to plaque assay to measure the virus titer. Statistical significance was determined using Student's t test. (C) The effect of RyDEN expression on the intracellular replication of the DENV replicon. A549 cells harboring the DENV-2 subgenomic RNA replicon carrying the luciferase reporter gene were transfected with plasmid DNA expressing V5-RyDEN (gray bars) or V5-BAP (white bars). Cell lysates were subjected to luciferase assay 2 days after transfection. As a control experiment, the siRNA duplex against DENV NS3 (or a scrambled siRNA duplex) was transfected (black bars). Luciferase activity in the cell lysate was normalized to total protein concentration. Statistical significance was determined using two-way ANOVA. (D) The kinetics of DENV RNA synthesis in RyDEN-expressing cells. V5-RyDEN (gray bars) and V5-DHFR (white bars)-expressing Huh7.5 cells were infected with DENV-2 at an MOI of 2, and RNA was isolated 6, 12, 18, and 24 h after infection. The copy numbers of total (T) and negative-strand (N) DENV RNA in 10 μg RNA samples were measured by qRT-PCR using random primer or 3’UTR-specific forward primer for RT step, respectively. Statistical significance was determined using two-way ANOVA.

To gain insight into the process of DENV replication that is affected by RyDEN, we first assessed the efficiency of virus entry using previously reported entry assays [34,40]. V5-tagged RyDEN or control protein (*Renilla* luciferase [RLuc])-expressing Huh7.5 cells were exposed to DENV-2 at an MOI of 5 at 37°C for 2 h, which allowed binding and internalization of virions, and then treated with a high-salt concentration alkaline solution on ice to remove uninternalized viruses, followed by additional washing with PBS. qRT-PCR analysis targeted against the DENV 3’UTR to measure the amount of internalized viruses showed that RyDEN expression did not influence the virus binding/entry process (Fig 5A).

To confirm the observation that the post-entry process is affected by RyDEN, an indirect assay, in which viral genomic RNA was transfected to cells to bypass the binding, entry, and uncoating steps of DENV replication, was performed [34,41]. Naked viral RNA was purified from a culture supernatant that contained infectious DENV-2 and was transfected to V5-RyDEN or control protein-expressing Huh7.5 cells. When infectious titers of DENV produced from transfected cells were analyzed by plaque assay 3 days after transfection, the production and subsequent replication of DENV was significantly inhibited by the expression RyDEN (Fig 5B).

Next, we examined the inhibitory effect of RyDEN on intracellular events in DENV replication using a reporter luciferase-expressing DENV-2 subgenomic RNA replicon system (DENrepPAC2A-Rluc [42]). The transient transfection of V5-RyDEN-expressing plasmid DNA into A549 cells harboring the DENV replicon exhibited a significant and dose-dependent suppression of the luciferase activity as compared with the V5- BAP control-expressing plasmid transfection (Fig 5C). As indicated in previous studies [42,43], treatment with two antiviral components, small interference RNA (siRNA) against DENV-2 NS3 (siNS3, Fig 5C) and mycophenolic acid (MPA) that has been demonstrated to prevent viral RNA replication (S5 Fig), resulted in drastic reductions in the replicon signal. Although the inhibition of DENV replicon activity by a transfection of RyDEN was less effective when compared to the NS3 siRNA or MPA treatment, it was still comparable to the level of inhibition by type I IFN treatment (S5 Fig).

We further monitored the kinetics of DENV RNA accumulation in virus-infected cells. When total DENV RNA (T) was measured by qRT-PCR analysis using a random primer for RT, a slight and insignificant decrease in the amount of viral RNA was detected in RyDEN-expressing cells 6 h after infection (2.3 times lower than in control protein-expressing cells, Fig 5D). Nevertheless, further and significant reductions in the level of total viral RNA were observed 18 and 24 h after infection (Fig 5D). When the level of negative-strand DENV RNA (N) as measured by qRT-PCR analysis using 3’UTR-specfic forward primer for RT was compared, a significant decrease in the amount of negative-strand RNA was also detected 18 and 24 h after infection in RyDEN-expressing cells (Fig 5D). However, the kinetics of accumulating total and negative-strand DENV RNA in the V5-RyDEN-expressing cells appeared to be similar to those in control cells (Fig 5D). Taken together, these data suggest that RyDEN somehow inhibits intracellular events of DENV replication independent of the entry, uncoating, assembly, or negative-strand RNA synthesis of a virus.

### RyDEN interacts with cellular mRNA-binding proteins that facilitate DENV replication

**Fig 6:**
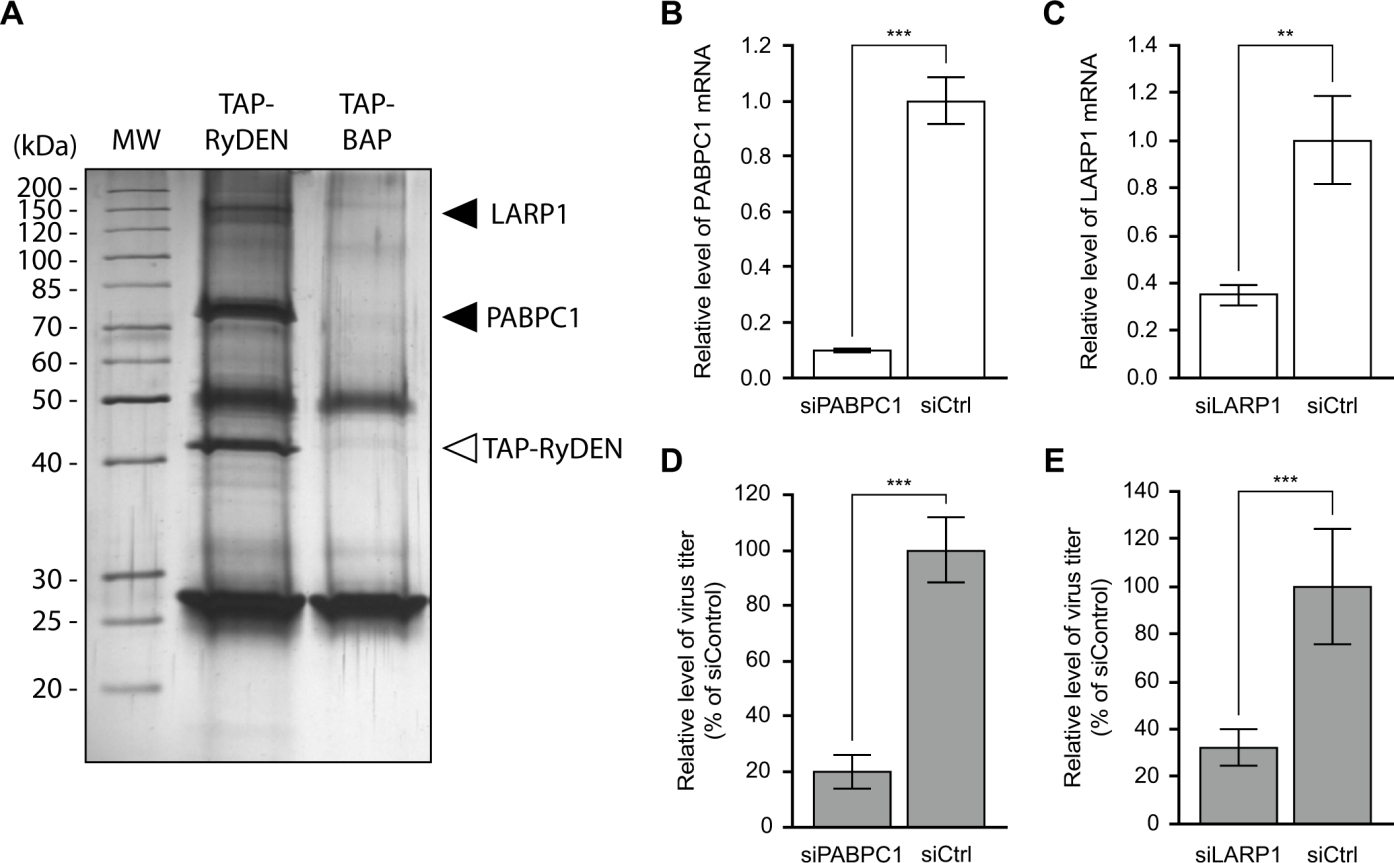
Interaction of RyDEN with PABPC1 and LARP1. (A) Affinity purification analysis for RyDEN. TAP tag-fused RyDEN (or BAP control protein) was expressed in HepG2 cells by lentiviral vector transduction, and protein complexes containing the TAP-RyDEN (or TAP-BAP) were isolated from cell lysates using IgG Sepharose beads. Purified proteins were visualized by silver staining on an SDS-PAGE gel and analyzed by MALDI TOF-TOF MS. MW, molecular weight marker. Note that protein band of TAP-BAP was overlapped with that of IgG heavy chain, both which were detected as 50 kDa bands in the gel. (B, C, D, and E) HepG2 cells were transfected with siRNA duplex against PABPC1 (siPABPC1, B and D) and LARP1 (siLARP1, C and E) or nonspecific siRNA duplex (siCtrl). Two days after transfection, cells were infected with DENV-2 at an MOI of 1. Two more days after infection, cells and culture supernatants were harvested for total RNA isolation (B and C) and plaque assay to quantify the virus titer (D and E), respectively. Levels of PABPC1 and LARP1 mRNA quantified by qRT-PCR were normalized with GAPDH mRNA levels. Statistical significance was determined by Student’s *t* test.

In order to search for additional clues regarding the function of RyDEN, we attempted to identify the interacting partners of RyDEN using an affinity purification-mass spectrometry approach. For this purpose, HepG2 cell lines stably expressing RyDEN or control protein (BAP) were fused by lentiviral vector transduction with an N-terminal tandem affinity purification (TAP) tag that contained two IgG binding units [44]. TAP-fused RyDEN and its associated proteins were recovered from the extract of the HepG2 cell lines using IgG Sepharose beads under physiological conditions [44]. SDS-PAGE and subsequent silver staining analysis showed that TAP-RyDEN, but not the TAP-BAP, were specifically co-purified with a >70 kDa band (Fig 6A). Mass spectrometry analysis then identified the >70 kDa band as a poly(A)-binding protein cytoplasmic 1 (PABPC1). Likewise, as we shall see below, mass spectrometry analysis revealed that the additional protein band with a molecular weight mass of around 150 kDa was the La motif-related protein 1 (LARP1). The >40-kDa protein band was confirmed to be TAP-RyDEN (Fig 6A and S6 Fig).

PABPC1 belongs to the evolutionally conserved PABP family of proteins that bind the 3’ poly(A) tail of the mRNA and have multiple roles in translation and mRNA stability [45]. PABPC1 is expressed widely in a variety of human tissues [46] and is reported to have multiple roles in cytoplasmic mRNA function [47,48]. Interestingly, a previous biochemical study by Polacek *et al*. demonstrated that PABP binds to the DENV 3’UTR RNA *in vitro*, despite the lack of a poly(A) tail in the viral genome, suggesting the modulatory activity of PABP in DENV mRNA translation [49]. LARP1 is one of the La-motif related proteins that is a superfamily of RNA-binding factor, which is conserved in eukaryotes [50]. This RNA-binding protein was first identified in *Drosophila*, and is reported to be involved in spermatogenesis, embryogenesis, and cell cycle progression [51–53]. In mammalian cells, it has been demonstrated that LARP1 regulates cell division, apoptosis, and cell migration [54]. It should be noted that LARP1 is found in a complex of certain poly(A)-binding proteins and also interacts with PABPC1 in *Drosophila* and human cells [53–55].

To examine the role of these cellular mRNA-binding proteins in DENV infection, HepG2 cells were subjected to gene silencing by siRNA against PABPC1 and LARP1, resulting in 10.0- and 2.9-fold reductions in mRNA expression, respectively, as measured by qRT-PCR analysis (Fig 6B and 6C). When siRNA-transfected cells were infected with DENV-2 at an MOI of 1, we found that the level of virus replication was significantly decreased in both knockdown cells as compared with the replication in non-targeting control siRNA (siCtrl, Fig 6D and 6E), implying that PABPC1 and LARP1 positively impact DENV replication. Note that, at least in the PABPC1 siRNA-transfected cells, severe growth defects, which might lead to limited DENV replication, was not likely to be caused by the depletion of PABPC1 (S7 Fig).

### Domain of RyDEN required for its anti-DENV activity

**Fig 7:**
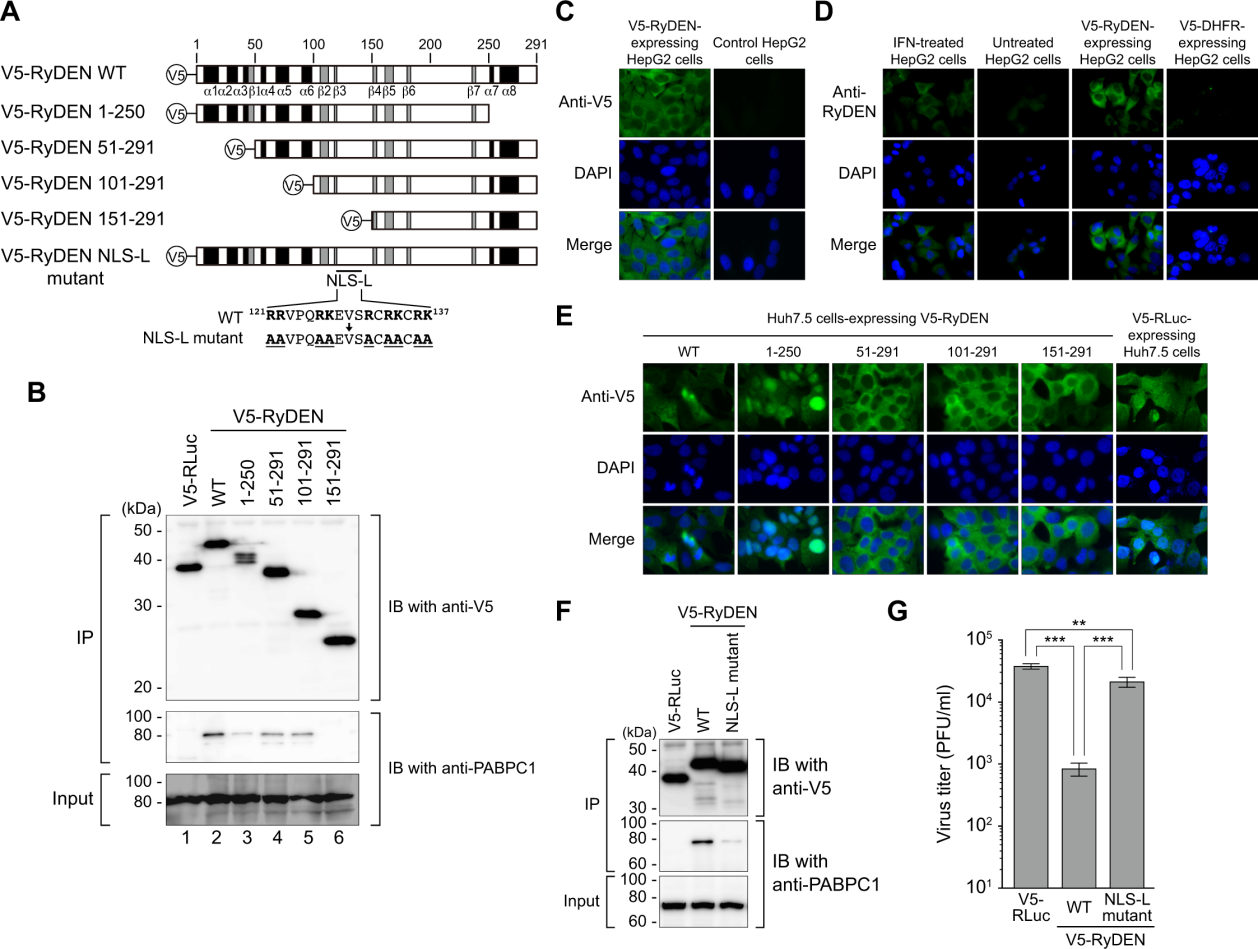
Requirement of PABPC1 interaction domain for anti-DENV activity of RyDEN. (A) Schematic of full-length (WT) RyDEN and its mutants used in experiments. N-terminal (51–291, 101–291, and 151–291) and C-terminal (1–250) deletion mutants and a site-directed mutant, in which arginine (R) and lysine (K) residues in a putative NLS sequence (NLS-L) were substituted with alanine (A), were constructed as V5-tagged proteins. Predicted α-helix (black) and β-sheet (gray) regions are also shown. (B) Mapping of the PABPC1-binding domain in RyDEN. A series of V5- RyDEN, including its truncation mutants (lanes 2–6) and V5-RLuc (control, lane 1), were lentivirally expressed in Huh7.5 cells, and cell lysates (input, bottom panel) were subjected to co-immunoprecipitation (IP) analysis using anti-V5 antibodies. Immunoprecipitates were then analyzed by immunoblotting (IB) using anti-V5 (for RyDEN and RLuc, top panel) or anti-PABPC1 (middle panel). Masses of molecular weight standards are indicated at left. (C) IFA of V5-protein expressing cells. HepG2 cells expressing V5-RyDEN or parental HepG2 cells were fixed with 4% paraformaldehyde (PFA), permeabilized with 0.1% Triton X-100, blocked with 5% goat serum, and stained with an anti-V5 antibody, followed by detection with Alexa Fluor 488-conjugated anti-mouse secondary antibody (top row). (D) IFA for endogenously expressed RyDEN. HepG2 cells that had been cultured in the presence or absence of IFN-α/ω (1,000 units/ml) for 24 h were fixed with PFA, permeabilized with 1% Triton X-100, blocked with Blocker Casein (Thermo Scientific), and stained with anti-RyDEN rabbit serum, followed by detection with FITC-conjugated anti-rabbit secondary antibody (top row). As another controls, V5-RyDEN- or V5-DHFR-expressing HepG2 cells was also stained with the anti-RyDEN serum (third and fourth columns). (E) Localization of RyDEN deletion mutants. Huh7.5 cells expressing V5-RyDEN (WT), its truncation mutants (1–250, 51–291, 101–291, 151–291), or control V5-RLuc were subjected to IFA using anti-V5 antibody. In all IFA, cell nuclei were stained with DAPI (center rows), and merged images are shown in the bottom rows. (F) HepG2 cells stably expressing V5- RyDEN (WT and NLS-L mutant) or V5-RLuc were generated by lentiviral vector transduction, and cell lysates (input) were used for immunoprecipitation analysis using anti-V5 antibodies. V5-tagged proteins and PABPC1 in the immunoprecipitates were detected by immunoblotting using anti-V5 (for RyDEN and RLuc, top panel) and anti-PABPC1 (middle panel) antibodies. (G) Activity of the RyDEN NLS-L mutant in the suppression of DENV replication. V5-tagged protein-expressing HepG2 cells were infected with DENV-2 at an MOI of 1, and culture supernatants were subjected to plaque assay 2 days after infection to measure the virus titer. Statistical significance was determined by one-way ANOVA with Dunnett’s multiple comparison test.

We sought to determine the RyDEN domain that is required for interaction with PABPC1. To map the binding domain, a series of V5-tagged RyDEN containing N- and C-terminally truncated mutants was constructed (Fig 7A) and stably expressed in Huh7.5 cells. In this experiment, V5-tagged RLuc was used as a control protein to confirm the specificity of RyDEN-PABPC1 interaction. Co-immunoprecipitation using anti-V5 antibodies followed by immunoblotting using anti-PABPC1 antibodies confirmed that full-length (WT) RyDEN interacted with PABPC1 (Fig 7B, middle panel, lane 2). Importantly, interaction with PABPC1 was also detected with RyDEN-truncated mutants 1–250, 51–291, and 101–291 (lanes 3–5), whereas RyDEN mutant 151–291 was not co-precipitated with PABPC1 (lane 6). Therefore, this result indicates that the domain of interaction with PABPC1 is located in RyDEN’s middle region, which is between amino acid positions 102–150.

As described above, RyDEN was predicted to possesses a sequence resembling a bipartite NLS (^121^RRVPQRKEVSRCRKCRK^137^, Fig 1F), which was called an NLS-like (NLS-L) sequence in this study. An IFA using V5-RyDEN-exppressing HepG2 cells and anti-V5 antibodies showed that a higher concentration of ectopically expressed RyDEN was found in cytoplasm (Fig 7C). When intracellular distribution of RyDEN was analyzed by IFA using a newly generated anti-RyDEN rabbit serum, endogenous RyDEN that had been induced by type I IFN localized mainly in the cytoplasm of HepG2 cells (Fig 7D). This cytoplasmic localization of RyDEN was observed in several other human cell lines as well (S8 Fig). By contrast, a parallel IFA using V5-RyDEN truncation mutant (Fig 7A)-expressing cells revealed that the localization of RyDEN to the nucleus was only observed when the C-terminal domain encompassing the putative NES sequence was deleted (V5-RyDEN 1–250, Fig 7E). These data indicate that, in the presence of C-terminal NES, the NLS-L sequence may not function as an active NLS to accumulate RyDEN in the nucleus.

Meanwhile, since the NLS-L sequence is located in the domain of RyDEN’s interaction with PABPC1 (102–150, Fig 7A), we examined whether the NLS-L mutations influenced the binding of RyDEN to PABPC1. To this end, we constructed a site-directed mutant of RyDEN, in which positively charged arginine (R121, R122, R126, R131, R133, and R136) and lysine (K127, K134, and K137) residues in NLS-L were changed to alanine (^121^AAVPQAAEVSACAACAA^137^, Fig 7A). Intriguingly, immunoprecipitation analysis using lysates of V5-tagged WT or NLS-L mutant RyDEN-expressing HepG2 cells revealed that the binding efficiency of RyDEN to PABPC1 was decreased by the mutation of NLS-L (Fig 7F). More importantly, when the replication of DENV-2 in each cell line was compared, although some inhibition of virus replication was still observed in the NLS-L mutant-expressing cells, its inhibitory effect was 25.4-fold lower than that obtained in WT RyDEN-expressing cells (Fig 7G). These results suggest that interaction with PABPC1 participates in RyDEN’s anti-DENV activity.

### Association of RyDEN with DENV RNA

**Fig 8:**
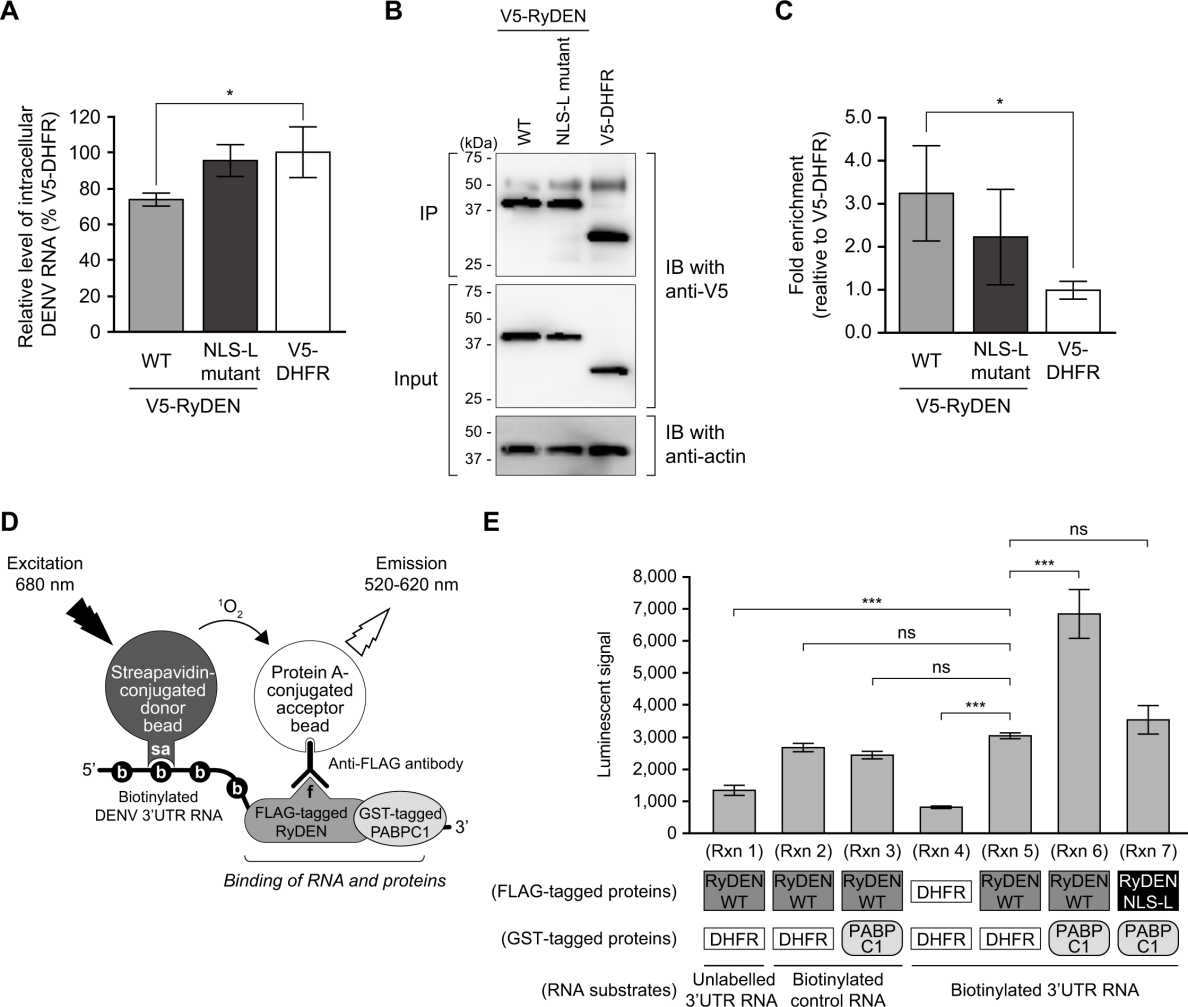
Association of RyDEN with DENV RNA. (A) HepG2 cells expressing V5-RyDEN (WT and NLS-L mutant) or V5-DHFR (control) were infected with DENV-2 at an MOI of 5, and 6 h after infection, cell lysates were subjected to RIP assay using anti-V5 antibodies. A portion of the cell lysates were used for total RNA extraction, and DENV RNA was quantified by qRT-PCR, which was normalized with GAPDH mRNA. (B) Immunoblotting analysis to detect V5-tagged proteins. Portions of the immunoprecipitated (IP, top panel) and input (middle and bottom panels) fractions were subjected to immunoblotting (IB) analysis using anti-V5 (for RyDEN and RLuc, top and middle panels) and anti-actin (bottom panel) antibodies. (C) Level of DENV RNA in immunoprecipitates. Total RNA was extracted from immunoprecipitated samples, and the DENV RNA level was analyzed by qRT-PCR. (D) Schematic diagram of AlphaScreen assay to detect the binding of RyDEN and DENV 3’UTR RNA. FLAG-tagged RyDEN produced by the wheat germ cell-free system was incubated with biotin-labelled (biotinylated) DENV-2 3’UTR RNA in the presence of GST-tagged proteins. RyDEN and 3’UTR RNA interaction bridges the streptavidin (sa)-coated donor bead and anti-FLAG-conjugated acceptor bead via recognition of biotin (b) of RNA and N-terminal FLAG-tag (f) of protein, respectively. Upon excitation at 680 nm, single oxygen molecules (^1^O_2_) are produced from the donor beads, which react with the acceptor beads, resulting in light emission measured between 520 and 620 nm (AlphaScreen signals). (E) *In vitro* interaction of RyDEN and DENV RNA. The AlphaScreen-based RNA-binding assay was performed with 20 nM FLAG-tagged proteins (RyDEN WT [Rxns 1, 2, 3, 5, and 6], RyDEN NLS-L mutant [Rxn 7], or DHFR [Rxn 4]) and 3.5 ng/μl substrate RNA (unlabeled DENV 3'UTR [Rxn 1], biotinylated control [derived from DHFR gene, 480 base, Rxns 2 and 3], or biotinylated 3'UTR [Rxns 4–7] RNA) in the presence of 20 nM GST-tagged proteins (RABPC1 [Rxns 3, 6, and 7] or DHFR [Rxns 1, 2, 4, and 5]). Statistical significance was determined by one-way ANOVA with Dunnett’s multiple comparison test. ns, no significance.

The functional interaction of RyDEN with cellular mRNA-binding proteins PABPC1 and LARP1 (Figs 6 and 7) prompted us to test whether RyDEN was recruited to DENV RNA during infection. To analyze the association of RyDEN with DENV RNA, RNA immunoprecipitation (RIP) assay was performed. HepG2 cells stably expressing V5-tagged protein were infected with DENV-2 at an MOI of 5, and cell lysates were subjected to immunoprecipitation 6 h after infection. In this experiment, we needed to harvest infected cells at an early time point to recover sufficient amounts of DENV RNA because, at a later time point, the amount of viral RNA synthesis had been shown to be dramatically inhibited by the overexpression of V5-RyDEN (Fig 5D). In fact, a significant reduction in the amount of DENV RNA was already detected in the input fraction of V5-RyDEN-expressing cells 6 h after infection (Fig 8A). Note that a significant reduction of viral RNA was not observed in NLS-L mutant RyDEN-expressing cells (a 27% reduction relative to V5-DHFR-expressing cells, Fig 8A). Cell lysates from V5-tagged WT RyDEN-, NLS-L mutant RyDEN-, and control DHFR-expressing cells were used for immunoprecipitation using anti-V5 antibodies, and the total RNA extracted from immunoprecipitates was detected by qRT-PCR analysis against the DENV-2 3’UTR. When the input fraction and the immunoprecipitates were subjected to an immunoblotting analysis, comparable levels of V5-tagged proteins were found to be pulled down by the immunoprecipitation (Fig 8B). However, as anticipated, immunoprecipitation with V5-RyDEN significantly enriched DENV RNA as compared to the level of viral RNA detected in V5-DHFR immunoprecipitates (Fig 8C). In contrast, immunoprecipitation with the NLS-L mutant of RyDEN, which was not able to impair DENV RNA synthesis 6 h after infection (Fig 8A) exhibited only slight enrichment of viral RNA (not statistically significantly different from the V5-DHFR sample, Fig 8C).

To further examine the association of RyDEN with DENV RNA, we performed an *in vitro* RNA-binding assay based on AlphaScreen technology (PerkinElmer). For this experiment, recombinant proteins (RyDEN and PABPC1) were obtained by the wheat germ cell-free protein production system, a eukaryotic cell-based *in vitro* translation method that allows the generation of properly folded high-quality proteins [56], because the expression of RyDEN in *E*. *coli* was found to be toxic to the bacterial cells. N-terminal FLAG-tagged (RyDEN WT, RyDEN NLS-L mutant, and control DHFR) and glutathione S-transferase (GST)-tagged (PABPC1 and control DHFR) proteins were produced, affinity purified (S9 Fig), and mixed with biotin-labeled DENV-2 3’UTR RNA (450 base), followed by incubation with streptavidin-coated donor beads, anti-FLAG antibodies, and protein A-conjugated acceptor beads. If FLAG-RyDEN interacts with biotinylated 3’UTR RNA, the reaction bridges the donor and acceptor beads by recognizing the biotin of RNA and FLAG-tagged proteins, respectively, which in turn enables the generation of singlet oxygen (O_2_(^1^D_g_)) from donor beads upon the illumination and the chemical energy transfer to acceptor beads, resulting in a luminescent AlphaScreen signal (Fig 8D) [56]. As shown in Fig 8E, a reaction containing FLAG-RyDEN WT and unlabeled (i.e. non-biotinylated) DENV 3’UTR RNA (Rxn 1) or FLAG-DHFR and biotinylated 3’UTR RNA (Rxn 4) gave a negligible background signal in the AlphaScreen assay. When FLAG-RyDEN WT was incubated with biotinylated 3’UTR RNA (Rxn 5), a significantly increased luminescent signal was detected, while this was also observed in the incubation with biotinylated nonspecific control RNA (Rxn 2), indicating the RNA-binding property of RyDEN. However, the binding signal between WT RyDEN and biotinylated 3'UTR was significantly enhanced by the presence of GST-PABPC1 (Rxn 6). The specific interaction between RyDEN and DENV 3'UTR in this reaction was shown by a competition assay using unlabeled 3'UTR RNA as a competitor (S10 Fig). In contrast, the addition of GST-PABPC1 did not change the luminescent signal of FLAG-RyDEN WT and the biotinylated control RNA incubation (Rxn 3). More importantly, even in the presence of GST-PABPC1, the RyDEN NLS-L mutant (Rxn 7), which was shown to have reduced binding activity to PABPC1 (Fig 7F), did not generate the higher interaction signals with biotinylated 3’UTR RNA that were observed in the incubation with RyDEN WT (Rxn 6). These data, therefore, demonstrate that RyDEN is an RNA-binding protein, and binding specificity to DENV RNA is provided through a complex formation with PABPC1.

### Translational suppression of DENV RNA by RyDEN

**Fig 9:**
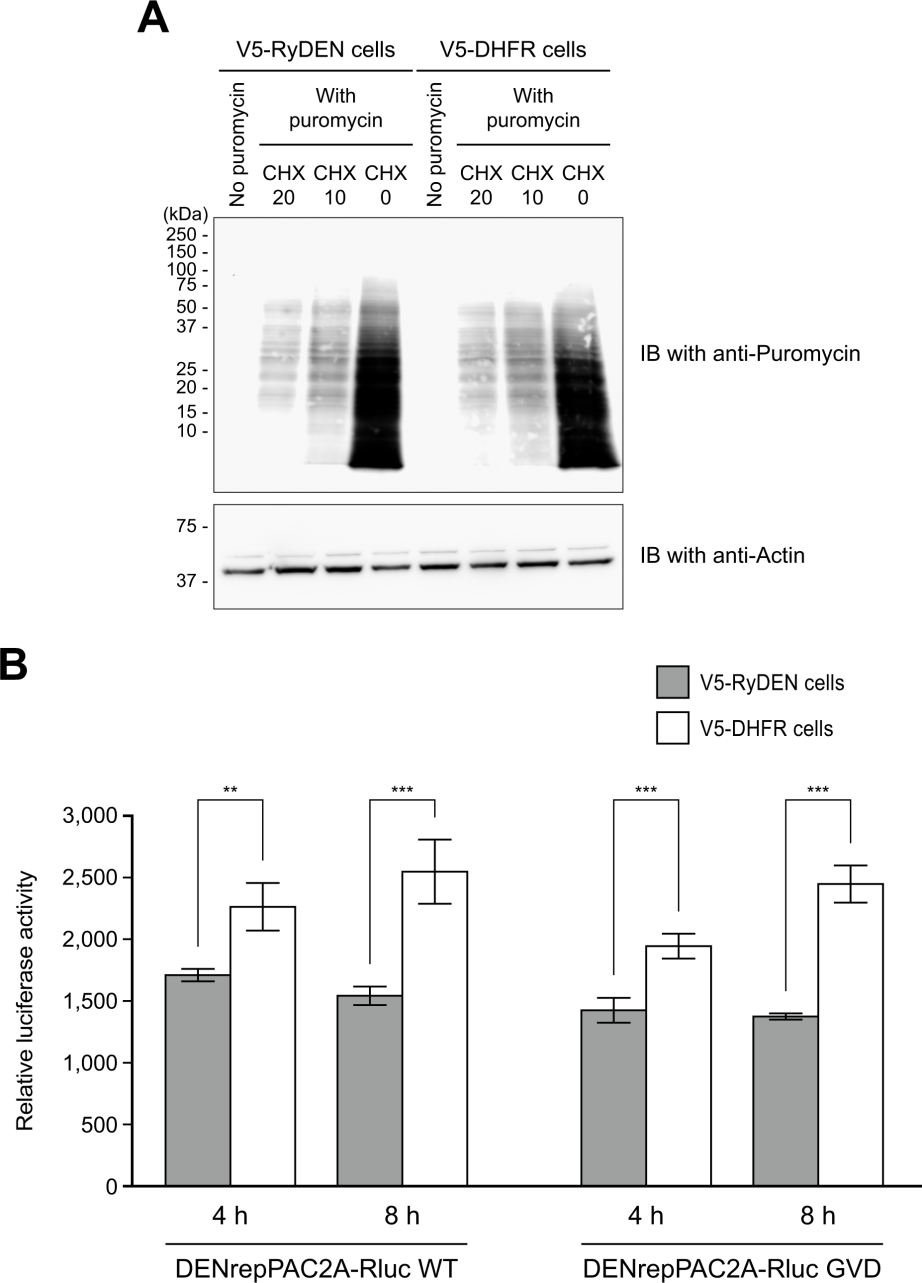
Decreased translation efficiency of DENV reporter constructs by RyDEN. (A) Puromycin labeling to monitor global protein synthesis. HepG2 cells expressing V5-RyDEN (left four lanes) or V5-DHFR (right four lanes) were cultured in the presence (20 μg/ml [CHX 20] and 10 μg/ml [CHX 10]) or absence (CHX 0) of cycloheximide. After 1 h incubation, 10 μg/ml of puromycin was added to the culture. Cells were harvested 40 min after puromycin pulse, and the cell lysate was subjected to immunoblotting (IB) using anti-puromycin antibody (top panel) and anti-actin antibody (bottom panel). (B) *In vitro* transcribed RNA of DENrepPAC2A-Rluc WT and its RdRp mutant, DENrepPAC2A-Rluc GVD, were transfected to V5-RyDEN (gray bars) or V5-DHFR (white bars)-expressing HepG2 cells, and cells were lysed and subjected to luciferase assay at 4 and 8 h after transfection. Luciferase activity in the cell lysate was normalized to total protein concentration. Statistical significance was determined by two-way ANOVA.

The interaction of RyDEN with PABPC1, an important molecule involved in cellular mRNA translation, led us to hypothesize that RyDEN might interfere with the translation process of DENV RNA. First, in order to investigate the effect of RyDEN expression on global cellular translation, puromycin labeling of newly synthesized proteins was performed using RyDEN-expressing cells [57]. HepG2 cells expressing V5-RyDEN or V5-DHFR were pulsed with puromycin, and cell lysates after the 40 min pulse were subjected to immunoblotting using anti-puromycin antibodies to compare the total protein synthesis of these two cell lines. As evident in control treatments in which cells had been treated with a protein synthesis inhibitor, cycloheximide, before puromycin pulse (CHX, Fig 9A), proteins detected by immunoblotting indicated *de novo* synthesized proteins that incorporated puromycin during mRNA translation in cells [57]. When the level of puromycin-labeled proteins was compared, there was no obvious difference in the protein synthesis of V5-RyDEN and V5-DHFR-expressing cells (Fig 9A), indicating that the global translation rate was not reduced by the expression of RyDEN.

We next examined the ability of RyDEN to interfere with protein synthesis from DENV RNA by employing a DENV-2-based luciferase reporter construct, DENrepPAC2A-Rluc [42]. DENV reporter RNA was transcribed *in vitro* transcribed using linearized construct DNA in the presence of an m7GpppA cap analogue and transfected to V5-RyDEN- or V5-DHFR-expressing HepG2 cells. As shown in Fig 9B, the RNA transfection of WT DENV reporter replicon (DENrepPAC2A-Rluc WT) exhibited reduced luciferase activity in V5-RyDEN-expressing cells when compared to V5-DHFR-expressing control cells 4 and 8 h after transfection. Importantly, diminished luciferase activity in the RyDEN-expressing cells at the early time points were also observed by RNA transfection of a mutant DENV reporter construct, DENrepPAC2A-Rluc GVD, in which the GDD motif in the active site of the RNA-dependent RNA polymerase (RdRp) had been changed to GVD (Fig 9B) [58]. Since the GVD mutation in the NS5 RdRp is reported to abolish viral RNA replication [58], the luciferase activity was considered to reflect the level of protein production from mRNA of the transfected construct. Although the inhibitory effect of RyDEN on the reporter protein production was relatively modest as compared to the inhibition levels observed in DENV replication (Fig 1) and viral RNA accumulation (Fig 5D), these data suggest that the expression of RyDEN is likely to be suppressive to the translation process of DENV RNA.

### Inhibition of a diverse range of viruses by RyDEN

**Fig 10:**
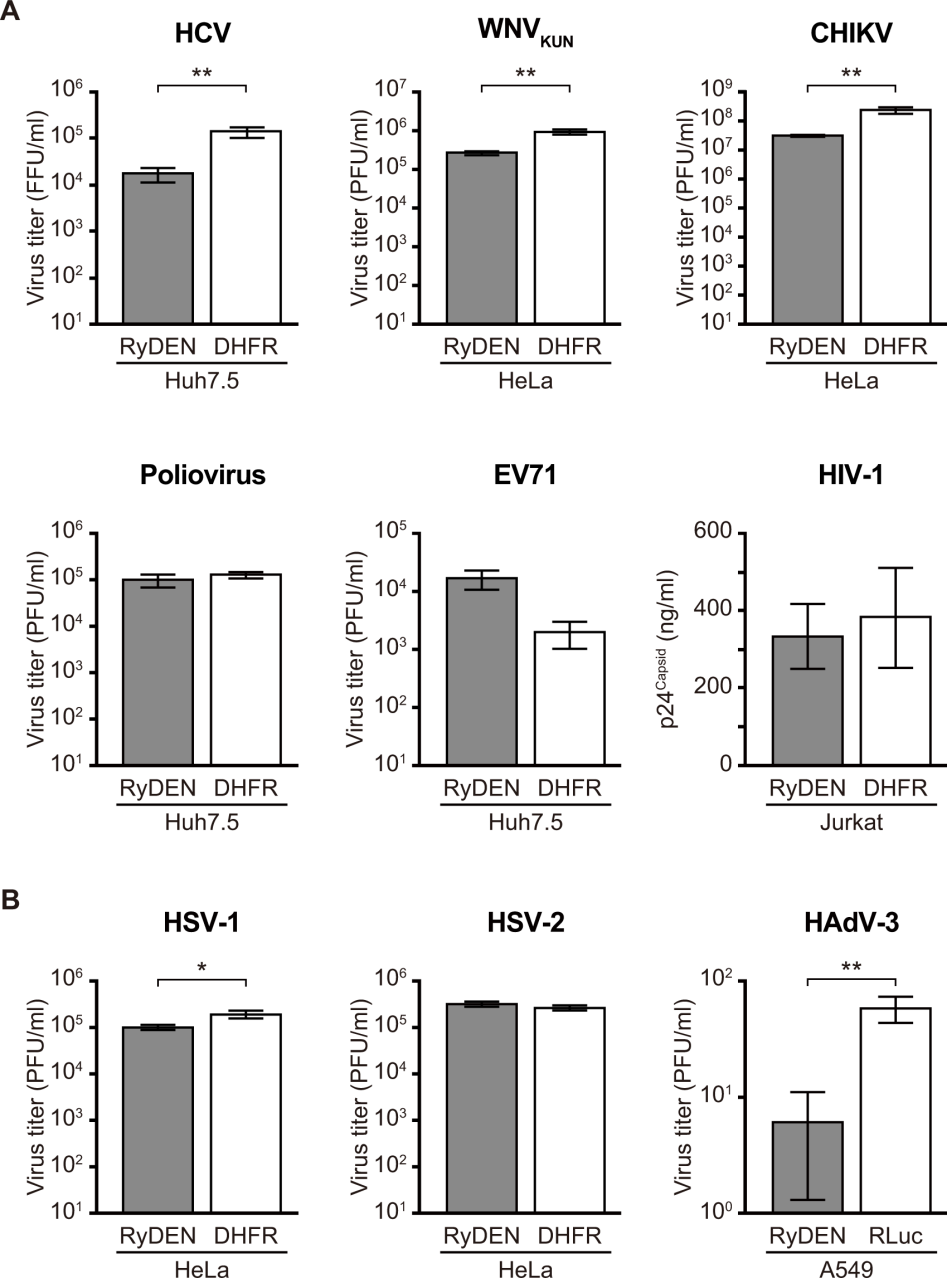
Effect of RyDEN expression on a diverse range of virus replications. Human cell lines (Huh7.5, HeLa, Jurkat, or A549) expressing V5-RyDEN (gray bars) and control proteins (DHFR or RLuc, white bars) were infected with six RNA (A) and three DNA (B) viruses at an MOI of 1, except for HIV-1, which was used at an MOI of 0.005. Levels of virus replications were analyzed using culture supernatants collected 1 day (poliovirus, EV71, and HAdV-3), 2 days (KUNV), 3 days (CHIKV, HSV-1, and HSV-2), 4 days (HCV), or 10 days (HIV-1) after infection by plaque assay, except for HCV (by IFA) and HIV-1 (by p24^Capid^ ELISA). FFU, focus forming units; PFU, plaque forming units. Statistical significance was determined by Student’s *t* test.

Since RyDEN was found to be involved in establishing an antiviral state against DENV in human cells, we also investigated whether the expression of RyDEN influences the replication of other viruses. To this end, V5-RyDEN-expressing cells were further created using human cell lines including HeLa, Jurkat, and A549 cells by lentiviral vector-mediated transduction. Cell lines were then infected with several RNA (hepatitis C virus [HCV, *Flaviviridae*], West Nile virus Kunjin strain [WNV_KUN_, *Flaviviridae*], Chikungunya virus [CHIKV, *Togaviridae*], poliovirus [*Picornaviridae*], human enterovirus 71 [EV71, *Picornaviridae*], and human immunodeficiency virus type-1 [HIV-1, *Retroviridae*]) and DNA (herpes simplex virus 1 [HSV-1, *Herpesviridae*], HSV-2, and human adenovirus type 3 [HAdV-3, *Adenoviridae*]) viruses. Measurements of the virus titer in the supernatants of infected cells indicated that significant inhibition by the overexpression of RyDEN was observed in HCV, WNV_KUN_, and CHIKV, but not in poliovirus, EV71, or HIV-1 infections, as compared to that in control protein-expressing cells (Fig 10A). A preliminary result showed that replication of the Sindbis virus, a *Togaviridae* family virus, was also suppressed in V5-RyDEN-expressing HeLa cells (S11 Fig), suggesting that *Flaviviridae* and *Togaviridae* family members are broadly susceptible to RyDEN. Intriguingly, the replication of some DNA viruses, including HSV-1 and HAdV-3, were negatively affected by V5-RyDEN expression, whereas it had no influence on HSV-2 infection (Fig 10B).

## Discussion

In terms of morbidity and mortality, dengue has emerged as one of the most important arthropod-borne diseases in the world, with cases predominantly documented in tropical and sub-tropical urban centers. Currently, the development of new antiviral medications and vaccinations against DENV is an urgently needed. In this regard, understanding the host innate immune response that restricts DENV replication, such as the IFN response, will be important for the development of antiviral agents and effective vaccines. In this study, we present RyDEN (C19orf66) as an ISG that limits all serotypes of DENV. Our findings suggest that RyDEN may target the translation of DENV RNA through interaction with other cellular RNA-binding proteins.

Expression cloning of the cDNA library is a powerful approach to the functional and comprehensive analysis of cellular genes; such a gain-of-function screen has been applied to identify host factors involved in DENV replication [33,59]. In this study, a library of cDNA was generated from mRNA of type I IFN-treated HeLa cells and lentivirally expressed in Huh7.5 cells that exhibited massive cell death with DENV infection (S1 Fig), which was expected to confer extensive resistance to DENV-induced cell death (Fig 1A). Indeed, one round of a DENV-2 challenge resulted in more than 50 surviving cell clones on a 150-mm dish. An additional infection assay showed that 32 clones remained more or less resistant to DENV infection (Fig 2). Sequencing analysis of cDNA recovered from DENV-resistant Huh7.5 cells revealed that 19 cells harbored the RyDEN gene. Although some of the cells also contained all or parts of other genes or non-ORF sequences, the full ORF of RyDEN was isolated from all cells (Fig 2), indicating that RyDEN should be a major determinant of resistance to DENV in a cDNA library screening assay. Intriguingly, almost the same mutant (amino acid position 304–702) of DNAJC14, an Hsp40 family member that has been identified as an anti-flavivirus factor by a cDNA library screen [60], was also recovered in this study (Fig 2, clone 31), demonstrating the integrity of our screening. The previous report by Yi *et al*. showed that despite screening using cDNA from IFN-α-treated cells, DNAJC14 mRNA levels were not upregulated by interferon treatment, although the DNAJC14 mutant was again identified with a cDNA library of IFN-treated HeLa cells in our study. Thus, it still would be interesting to investigate how the DNAJC14 function is associated with the IFN-mediated antiviral response. In addition, the future investigation of other genes identified in our IFN cDNA library screen (e.g., IFN-α-inducible protein 27, C19orf53) in flavivirus replication including DENV will provide fascinating insights into the interaction between virus and host.

RyDEN is expressed from chromosome 19 as an eight-exon gene that encodes a 291 amino acid protein (Fig 1E). A BLAST search analysis using RyDEN’s amino acid sequence did not show any overt similarities with other proteins in mammals; however, this protein was predicted to contain a zinc-ribbon domain in the central region and a coiled-coil motif in the C-terminal region (Fig 1F). The zinc-ribbon motif, which is basically defined by CXXC(H)-15/17-CXXC, is a general architectural motif initially found in some eukaryotic transcription factors and RNA polymerase subunits that currently form largest group of zinc fingers [37]. Although the zinc ribbons seem to display limited sequence similarities, structural analysis revealed that a variety of cellular and viral proteins possess this motif as a binding domain for zinc [37]. Of interest is the fact that cyclic GMP-AMP synthase (cGAS), a cytosolic DNA-recognition receptor for the induction of IFN responses, has recently been shown to contain zinc ribbon, which is likely to be required for DNA recognition [61]. Since zinc ribbon is found in many DNA- and RNA-binding proteins [37], RyDEN may harbor nucleic acid-binding activity, as discussed below. Also, in amino acid sequence-based protein motif prediction programs, putative NLS (referred to as NLS-L) and NES sequences were found in the zinc-ribbon (121–137) and C-terminal domains (261–269), respectively (Fig 1F). Since IFA experiments showed that RyDEN was mainly dispersed throughout the cytoplasm (Fig 7C and S8A Fig), at least in a normally dividing cell, the NLS-L sequence does not function to accumulate RyDEN in the nucleus. However, deletion of the C-terminal domain containing the putative NES sequence led to an exclusively nuclear location (Fig 7E), suggesting that RyDEN is a potential nucleocytoplasmic shuttling protein, which is mostly retained in the cytoplasm. Note that no obvious changes in the subcellular localization of overexpressed RyDEN were observed with IFN treatment or DENV infection (S8B Fig).

In this study, RyDEN was shown to be an antiviral ISG. The overexpression of RyDEN in human cells suppressed all serotypes of DENV (Fig 3C) and, importantly, the endogenous expression of RyDEN was upregulated with the treatment of types I, II, and III IFNs (Fig 4B). Although the level of artificially expressed RyDEN (i.e. V5-RyDEN) was 38.7±2.1 times higher than that of IFN-induced endogenous RyDEN in HepG2 cells as measured by qRT-PCR analysis, we believe that the expression level of IFN-induced RyDEN sufficiently participates in the inhibition of DENV replication in human cells. Supporting this, in the RyDEN knockdown cell line, the inhibitory effect of type I IFN against DENV-2 was reduced by more than 70% (Fig 4C), indicating a major contribution of RyDEN to the IFN-mediated anti-DENV response. It should also be noted that even without IFN treatment, knockdown of the endogenous expression of RyDEN significantly enhanced DENV replication in several cell lines (Fig 3E and S3 Fig), indicating that a steady-state level of RyDEN acts as a DENV inhibitor. In addition, expression levels of RyDEN as measured by qRT-PCR varied among different human cell lines (S4 Fig), RyDEN expression may be one intracellular factor that determines the cellular tropism of DENV.

One question to ponder is, how does RyDEN suppress the replication of DENV? When the efficiency of virus entry was assessed by qRT-PCR, the level of viral RNA internalized in RyDEN-expressing cells was comparable to that in the control cells (Fig 5A). In contrast, a significant decrease in the level of intracellular DENV RNA was observed in RyDEN-expressing cells 18–24 h after infection (Fig 5D). RyDEN was, therefore, suggested to inhibit the post entry process during DENV replication. Consistent with this, the use of a cell line that harbored the RLuc reporter gene-carrying DENV subgenomic RNA replicon showed that the suppression of luciferase activity occurred with the transient expression of RyDEN (Fig 5C) at a level similar to IFN treatment (S5 Fig). Importantly, transfection of a replication-defective mutant of the DENV reporter construct RNA (DENrepPAC2A-Rluc GVD [58]) showed that luciferase activity of the reporter construct RNA was diminished by the expression of RyDEN (Fig 9B). Since RyDEN was not a mediator of the IFN response (Fig 4), these results suggest that RyDEN is a downstream effector molecule in the anti-DENV IFN response, which may target the translation process of viral RNA. Nevertheless, when compared to more pronounced effect on DENV titers (Fig 1) and viral RNA levels (Fig 5D), the inhibitory effect of RyDEN on the protein translation was modest (Fig 9B). Therefore, we cannot rule out the possibility that RyDEN may also interfere with other step(s) of DENV replication such as RNA transcription or protein processing.

Affinity purification-mass spectrometry analysis using TAP-tagged RyDEN then provided an important clue about RyDEN’s mechanism-of-action: RyDEN was likely to form a complex with the cellular RNA-binding protein PABPC1 (Fig 6A). PABPC1 is one of the major PABP-family proteins in eukaryotes and is ubiquitously expressed in cytoplasm [45]. Although PABPC1 is reported to play multiple roles in the translation, deadenylation, and stability of mRNA through binding to a 3’ poly(A) tail, the typical function of this protein is to form the closed-loop structure of mRNA by interaction with eIF4G, a subunit of the 5’ cap-binding eIF4E complex, to initiate protein translation [47,48]. Of particular interest, a previous study by Polacek *et al*. has shown that PABP is able to bind the 3’UTR of DENV *in vitro* [49]. Although the DENV RNA genome lacks a terminal Poly(A) tail, Polacek *et al*. reported that A-rich stretches upstream of the stem-loop in the 3’UTR appeared to be involved in PABP binding [49]. In our study, the interaction domain of RyDEN with PABPC1 was mapped to the central region between amino acid positions 102–150 (Fig 7A and 7B). Importantly, alanine substitution of positively charged arginine and lysine residues in the NLS-L sequence (121–137) of RyDEN resulted in decreased efficiency in the interaction with PABPC1 and reduced inhibitory activity against DENV replication (Fig 7G).

Additionally, the affinity purification-mass spectrometry analysis identified LARP1 as another interactor with RyDEN (Fig 6A). LARP1 is also an RNA-binding protein that contains two RNA-binding motifs called the La motif and the RNA recognition motif [50]. While it has been documented that the La motif-related protein family is involved in a broad range of activities in cellular RNA, including tRNA processing and mRNA metabolism, LARPs are also reported to affect the translation process of mRNA [50]. In fact, it has been shown that LARP1 associates with PABPC1 and eIF4E in human cells and has a positive role at an early stage of translation initiation [54]. In our study, PABPC1 and LARP1 were found to be positive regulators of DENV, since the siRNA-mediated knockdown of these genes significantly reduced the level of virus replication in HepG2 cells (Fig 6). Given the fact that both PABPC1 and LARP1 have RNA-binding activity [45,50], one could envisage that RyDEN may associate with DENV RNA through its interaction with these proteins during infection. As expected, our data of RIP assay showed that DENV RNA was significantly enriched by V5-tagged RyDEN (Fig 8C). Moreover, AlphaScreen technology-based *in vitro* RNA-binding assay revealed that RyDEN possessed binding activity to DENV 3’UTR RNA, and the association of RyDEN with 3’UTR RNA was enhanced by the presence of PABPC1 (Fig 8E).

**Fig 11:**
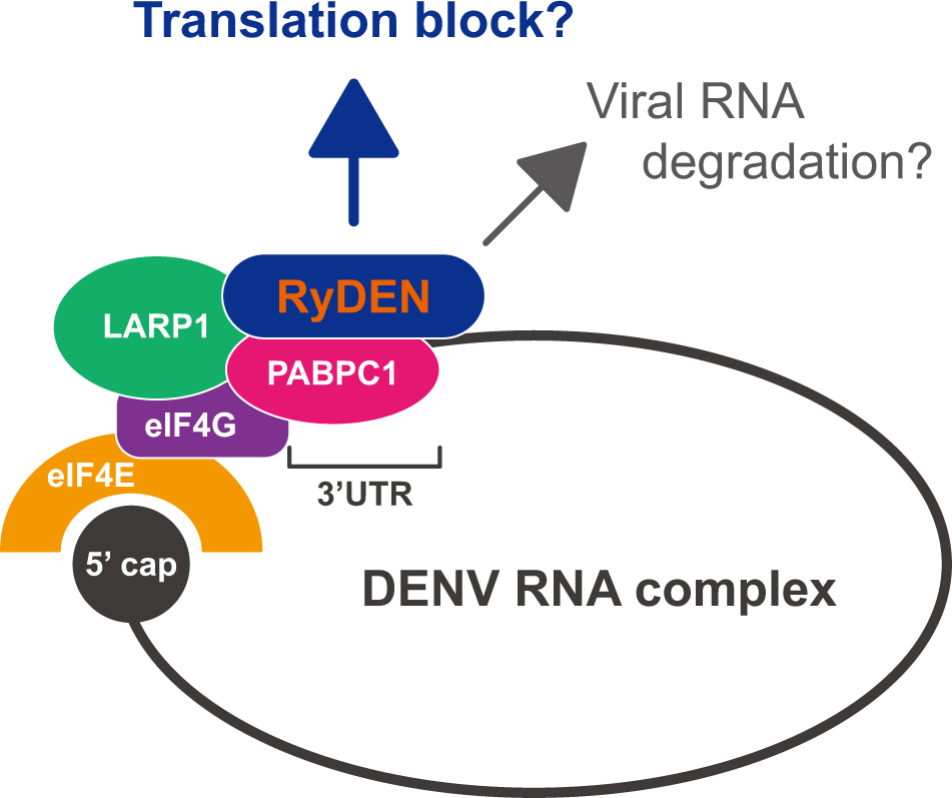
Possible models for RyDEN’s mechanism of action in the suppression of DENV infection. During DENV replication, PABPC1 and LARP1 are recruited to viral RNA, form a closed-loop structure of viral RNA with a cap-binding complex that includes eIF4G and eIF4E, and serve as positive regulators for the translation of viral proteins. RyDEN, whose expression is upregulated by IFN, specifically recognizes the DENV translation complex via interaction with viral RNA and PABPC1/LARP1. This interaction may interfere with the protein translation machinery of DENV RNA. Additionally, functions of PABPC1 and LARP1 in the regulation of mRNA turnover may be enhanced by interaction with RyDEN, resulting in the degradation of viral RNA in cytoplasmic foci such as P-bodies.

Therefore, based on our findings and the reported functions of PABPC1/LARP1, the following possibility could be proposed regarding the mechanism of RyDEN-mediated antiviral activity in DENV-infected cells: RyDEN forms a complex with PABPC1 (and LARP1) on DENV RNA, and then somehow interferes with the translation machinery of circularized viral RNA (Fig 11). This scenario would be consistent with the previous report by Diamond and Harris, in which IFN treatment was shown to inhibit the translation of DENV RNA rather than by preventing the association of DENV RNA with ribosomes [34]. In light of the data obtained by *in vitro* RNA-binding assay (Fig 8E), one could envisage that RyDEN’s RNA-binding activity is RNA sequence-nonspecific, but that it gains specificity to positive-strand DENV RNA via interaction with PABPC1 that has been suggested to recognize A-rich stretches in the 3’UTR [49]. Intriguingly, Paip2, a suppressor of PABPC1, has been reported to be such a cellular inhibitor in the viral translation machineries [49,62]. The above-mentioned study of Polacek *et al*. also presented fascinating evidence that Paip2 is able to block the interaction of PABPC1 with DENV 3’UTR RNA *in vitro* [49]. Furthermore, a recent work has revealed that Paip2, whose expression is stimulated by human cytomegalovirus (HCMV) infection, limits HCMV protein synthesis and replication [62]. It is noteworthy that a characteristic glutamic acid (E)-rich domain that has been characterized as a binding domain of Paip2 to PABPC1 [63] was also found in the C-terminal region of RyDEN (Fig 1F). Although the C-terminal region surrounding the E-rich domain of RyDEN appeared not to be critical to its interaction with PABPC1 (Fig 7B), RyDEN and Paip2 may have evolutionarily gained a similar regulatory function controlling PABPC1 activity. One concern would be that the translational suppression by RyDEN through interaction with PABPC1 might lead to a translation arrest of the host cell, which would result in the suppression of DENV replication. However, global cellular protein synthesis was not inhibited by the overexpression of RyDEN (Fig 9A). It is therefore conceivable that there is specificity to RyDEN’s recognition of the viral RNA translation complex. In addition, RyDEN may stimulate the degradation of DENV RNA in cytoplasmic P-bodies or stress granules (SGs) in collaboration with PABPC1 and/or LARP1, since the role of PABPC1 and LARP1 in eukaryotic mRNA decay as P-body and SG components has also been demonstrated [64,65]. These should be interesting topics to address in the future.

Our study has also shown that multiple viruses are susceptible to the inhibitory action of RyDEN to a greater or lesser extent, including HCV, WNV_KUN_, and CHIKV, whereas the replication of other RNA viruses tested (poliovirus, EV71, and HIV-1) was not suppressed by RyDEN overexpression (Fig 10A). Interestingly, some DNA virus replications (HSV-1 and HAdV-3) were also affected by RyDEN (Fig 10B). Our preliminary data showed that the replication of the Sindbis virus was impaired in V5-RyDEN-expressing cells (S11 Fig), suggesting that RyDEN acts as a broad-ranging inhibitory factor, at least against the *Flaviviridae* and *Togaviridae* families. Given the proposed model of RyDEN’s inhibitory mode of action against DENV (Fig 11), viruses whose replication is influenced by RyDEN may utilize PABPC1/LARP1 in their replication, particularly in the viral protein translation process. It should be emphasized that PABPs are well-known targets of several viruses, and it has been demonstrated that enteroviruses and lentiviruses cleave PABP by their protease to shut off cellular translation; in contrast, an HSV-1 protein binds PABP to stimulate viral mRNA translation [66]. Therefore, we hypothesize that the antiviral activity of RyDEN depends on whether the virus requires PABPC1 (and LARP1) function in its replication cycle. Indeed, PABPC1 is shown to promote HCV infection [67], which was inhibited by RyDEN (Fig 10A). In agreement with our data, a recent comprehensive study by Schoggins *et al*. using an overexpression screening of an ISG library has also reported the anti-HCV activity of RyDEN (shown as FLJ11286 gene [24]). Thus, further understanding of the molecular detail of RyDEN will contribute to the development of broadly active antiviral inhibitors.

## Materials and Methods

### Cells and viruses

HEK293T (human embryonic kidney, American Type Culture Collection [ATCC] CRL-11268), Huh7.5 (human hepatocellular carcinoma [68], obtained from Apath, LLC), HepG2 (human hepatoma, ATCC HB-8065), and HeLa (human cervical carcinoma, ATCC CCL-2) cells were cultured in DMEM supplemented with 10% fetal calf serum (FCS, Life Technologies) and antibiotics (100 units/ml penicillin and 100 μg/ml streptomycin). A549 (human lung adenocarcinoma, ATCC CCL-185) and Vero (green monkey kidney, ATCC CCL-81) cells were maintained in F-12K and Eagle's Minimum Essential Medium, respectively, which were supplemented with 10% FCS and antibiotics. BHK-21 (baby hamster kidney, ATCC CCL-10) and Jurkat (human lymphoblastoid T, ATCC TIB-152) cells were grown in RPMI 1640 supplemented with 10% FCS and antibiotics. C6/36 (*Aedes albopictus* mosquito, ATCC CRL-1660) cells were maintained at 28°C in HEPES-modified RPMI 1640 containing 10% FCS and antibiotics.

The four serotypes of DENV, which was isolated from isolated from patients recruited into the EDEN (early dengue infection and outcome) study in Singapore (DENV-1: Singapore isolate S144; DENV-2: Singapore isolate EDEN2 3295; DENV-3: Singapore isolate EDEN 130/05; and DENV-4: Singapore isolate S8976 [36,41]), DENV-2 (New Guinea C strain), CHIKV (Ross strain), and WNV_KUN_ were propagated in the C6/36 mosquito cells, and viral infectivity was titrated by plaque assays using BHK-21 cells as described previously [30]. HCV J6/JFH1-P47 (genotype 2) was produced using Huh7.5 cells and the virus titer was determined as focus forming units (FFU)/ml by previously reported IFA [69] on Huh7.5 cells using mouse anti-HCV core monoclonal antibodies (MA1-080, Pierce). Poliovirus (Sabin strain) and human enterovirus 71 (Singapore isolate) were propagated in RD cells, and viral infectivity was titrated by plaque assays using RD cells. HIV-1 (NL4-3) was produced by a transfection of HEK293T cells with pNL4-3, and the virus titer of the culture supernatants collected was determined as previously described [35]. Production and titration of HSV-1/2 and HAdV-3 were carried out using Vero and A549 cells, respectively. Virus titer was calculated as plaque-forming units (PFU)/ml (except for HCV and HIV-1).

### Generation of lentiviral vectors carrying an IFN cDNA library

A Gateway-compatible cDNA library was generated from mRNA isolated from HeLa cells that had been treated with 1,000 U/ml type I IFN (a mixture of human interferon α and ω, Sigma) for 24 h. Briefly, total RNA was extracted using the RNeasy Mini Kit (Qiagen), and mRNA was then isolated using a PolyATtract mRNA Isolation System II (Promega) according to the manufacturer’s recommendations. The cDNA was synthesized using the CloneMiner cDNA Library Construction Kit (Life Technologies) from 3 μg of mRNA and fractionated with cDNA Size Fractionation Columns (Life Technologies). After BP recombination reaction (Life Technologies) using 100 ng of cDNA and 300 ng of an entry vector, pDONR221, the entry library containing approximately 2.5 x 10^7^ clones, was amplified as a pool of transformants in One Shot TOP10 Electrocomp *E*. *coli* cells (Life Technologies). The entry vector plasmid DNA was purified using the QIAGEN Plasmid Midi Kit (Qiagen). To generate the lentiviral vector cDNA library, LR recombination reaction (Life Technologies) was performed using 300 ng of the entry cDNA library and 300 ng of an *Eco*RI-digested destination vector, pYK005C [35]. The resultant vector library was amplified as a pool of recombinants in One Shot TOP10 Electrocomp *E*. *coli* cells and purified using the QIAGEN Plasmid Maxi Kit (Qiagen).

A VSV-G-pseudotyped lentiviral vector expressing the IFN cDNA library was produced by the calcium phosphate-mediated transfection method using HEK293T cells as described previously [35]. Concentrated lentiviral vectors were titrated with HEK293T cells by evaluating the percentage of humanized *Renilla* green fluorescence protein positive cells 48 h after infection using a CyAn ADP flow cytometer (Beckman Coulter).

### Isolation of DENV-resistant cells and identification of the cDNA

In a 150-mm dish, 1 x 10^7^ of Huh7.5 cells were seeded 1 day before transduction and infected with 5 x 10^6^ infectious dose of the IFN cDNA carrying lentiviral vectors for 24 h. After 48 h post-transduction, the cells were challenged with DENV-2 (EDEN2 3295) at an MOI of 1. The culture medium was changed every 2–3 days, and after 2 weeks, cell colonies that survived the DENV challenge were transferred to 48-well plates and expanded for further analysis.

Genomic DNA was isolated from the resistant clones using the Wizard Genomic DNA Purification Kit (Promega) from cells that displayed low infectivity of DENV in immunofluorescence and plaque assay. The cDNA was then amplified by PCR using KOD-Plus 2 DNA polymerase (Toyobo) and primers (5’-CTT CCA TTT CAG GTG TCG TGA ACA CGC TAC CGG TCT CGA G-3’ and 5’-CAA ACG CAC ACC GGC CTT ATT CCA AGC GGC TTC GGC CAG-3’) flanking the Gateway cassette in the pYK005c lentiviral vector. cDNA was further amplified by nested PCR using primers (5’-ACC GGT CTC GAG AAT TAT CAA CAA-3’ and 5’-GCT GCA GAA TTA TCA ACC ACT TTG-3’) and cloned into the pCR-Blunt II-TOPO vector (Life Technologies). The sequence of cDNA in the pCR-Blunt II-TOPO vector was analyzed by an automated DNA sequencer, and the data was compared with the DNA database at the National Center for Biotechnology Information using a BLAST search.

### IFA

To stain for DENV dsRNA in surviving clones, 3 x 10^4^ of cells preseeded in Lab-Tek II 8-well chamber slides (Thermo Scientific) were infected with DENV-2 at an MOI of 5. Two days after infection, cells were fixed with 4% PFA for 30 min, permeabilized with 0.1% Triton X-100 in PBS for 10 min, and blocked with 5% goat serum and 0.5% BSA in PBS for 30 min at room temperature. The cells were stained with anti-dsRNA mouse monoclonal antibody (J2, English & Scientific Consulting Bt.), followed by a secondary antibody, Alexa Fluor 488-conjugated goat anti-rabbit IgG (Life Technologies). A slide was mounted with a ProLong Gold antifade reagent containing DAPI (Life Technologies) and observed under an Olympus IX81 fluorescence microscope. Images were captured with the CellSens Dimension software (Olympus). Staining of V5-tagged proteins was performed using a primary antibody, anti-V5 mouse monoclonal (Life Technologies), followed by Alexa Fluor 488-conjugated anti-mouse secondary antibody (Life Technologies).

To detect endogenous RyDEN, a rabbit serum was generated by Sigma using synthesized 4 peptides derived from RyDEN (amino acid positions 1–19, 51–69, 186–205, and 223–242). Cells preseeded in 8-well chamber slides (3 x 10^4^ of cells per well) were incubated with 1,000 U/ml type I IFN for 24 h, fixed, permeabilized with 1% Triton X-100, and blocked with Blocker Casein (Thermo Scientific). Immunostaining was carried out by an incubation with anti-RyDEN rabbit serum (1:5,000 in blocking buffer) and subsequent incubation with FITC-conjugated donkey anti-rabbit IgG (Rockland).

### Establishment of stable cell lines

To create stable cell lines expressing V5-tagged proteins, the ORF of RyDEN and the control proteins (DHFR and RLuc) were amplified by PCR and cloned into pDONR221 through a Gateway BP reaction. The individual ORF was then transferred to a Gateway-compatible lentiviral vector, pYK-nV5-Bla, in which a V5 epitope tag sequence had been added to the upstream of the Gateway unit in pYK005C-Bla [70] by LR reaction. A VSV-G-pseudotyped lentiviral vector was produced as described above and used to transduce human cells, including Huh7.5, HepG2, HeLa, Jurkat, and A549 cells. Transduced cells were selected in the presence of 10 μg/ml of blasticidin (InvivoGen). Expressions of V5-tagged proteins in the stable cell lines were confirmed by immunoblotting using anti-V5 antibodies as described below.

To construct a lentiviral vector that expressed shRNA, synthesized oligonucleotides that contained shRNA sequences against RyDEN ORF (sh1425: 5’-GAA CTA AGT AAC GAT CTG GAT-3’; sh3151: 5’-GAG AAG TTT CAT GGG AAG GTA-3’; sh5890: 5’- GAA GCC AAC CTA CGC ATG TTT-3’) were designed by using the RNAi Consortium web portal (http://www.broadinstitute.org/rnai/public/) and inserted into *Age*I-*EcoR*I sites of a lentiviral vector pLKO.1 puro (Addgene). VSV-G-pseudotyped lentiviral vector particles were produced by the transfection of lentiviral vector DNA encoding sh1425, sh3151, sh5890, or non-targeting control shRNA (SHC002, Sigma) and used to transduce HeLa cells. Transduced cells were selected over 2 weeks with 2 μg/ml of puromycin (InvivoGen). The knockdown efficiency of RyDEN mRNA in each cell line was analyzed by qRT-PCR as described below. The shRNA-resistant RyDEN expression vector was constructed using pYK005C-Bla by replacing the sh1425-targeting sequence of 5’-GAG CTG AGC AAT GAC CTC GAC-3’, which introduced seven silent mutations without changing the amino acid sequences of RyDEN.

### qRT-PCR analysis

Total RNA was isolated from cells using the RNeasy Mini Kit (Qiagen) and was treated with DNase using the TURBO DNA-free Kit (Ambion). cDNA was synthesized using High-Capacity cDNA Reverse Transcription Kit (Applied Biosystems), and subjected to real-time qPCR using SsoAdvanced SYBR Green Supermix and CFX96 Real-Time PCR detection system (Bio-Rad). The expression levels of target RNA were calculated by the comparative cycle threshold (CT) method and normalized with GAPDH mRNA levels. In some experiments for the detection of DENV-2 RNA, qRT-PCR was performed by High-Capacity cDNA Reverse Transcription Kit and SsoFast Probes Supermix (Bio-Rad) using previously described primers and fluorescent probe targeting 3’UTR of the DENV genome [71]. For qRT-PCR analysis of DENV-2 minus-strand RNA, cDNA synthesis was carried out using forward primer of 3'UTR instead of random primer as described in previous report [72]. Primer sequences for qRT-PCR analysis are listed in S1 Table.

### Immunoblotting analysis

Protein samples were denatured in an SDS sample buffer, separated by 10% SDS-PAGE gel, and transferred to an Immobilon-P transfer membrane (Millipore). The primary antibodies used were anti-V5 mouse monoclonal (Life Technologies), anti-C19orf66 rabbit polyclonal (Abcam), anti-PABPC1 mouse monoclonal (10E10, Santa Cruz Biotechnology), anti-ISG15 rabbit polyclonal (2743, Cell Signaling), and anti-actin mouse monoclonal (AC40, Sigma) antibodies. Horseradish peroxidase (HRP)-conjugated anti-mouse or anti-rabbit IgG antibody (Cell Signaling) was used as a secondary antibody. For immunoprecipitation analysis, TrueBlot ULTRA anti-mouse IgG HRP (Rockland) was used as a secondary antibody. Proteins were detected using an ImageQuant LAS 4000 mini chemiluminescent image analyzer (GE Healthcare).

### IFN treatments

To analyze RyDEN expression, HeLa cells preseeded in a 12-well plate at 1 x 10^5^/well density 1 day before treatment were incubated with 10, 100, or 1,000 units/ml of IFN-α/ω at 37°C. Twenty-four hours after treatment, cells were collected and subjected to immunoblotting using an anti-RyDEN antibody. In a parallel experiment, HepG2 cells expressing sh1425 and shCtrl were treated with 300 units/ml of IFN-α/ω, IFN-γ (BioLegend), or IFN-λ1 (PeproTech) for 24 h before assessing RyDEN expression by immunoblotting. For DENV infection, shRNA-expressing HepG2 cells were treated with or without IFN-α/ω (300 units/ml) for 24 h and then inoculated with DENV-2 at an MOI of 1. The culture supernatant was collected 48 h after infection and subjected to plaque assay.

### Virus infection assays

For DENV infection, cells (V5-tagged protein or shRNA-expressing) preseeded in 6-well plate at 5 x 10^5^/well density 1 day prior to infection were infected at an MOI of 0.1, 1, or 10. After 1 h of incubation at 37°C, cells were washed once followed by replacement with growth medium without selection antibiotics. The culture supernatant was collected at indicated time points and subjected to a standard plaque assay. In a similar way, HCV, poliovirus, and EV71 infections were performed by exposing the viruses to V5-RyDEN or V5-DHFR-expressing Huh7.5 cells at an MOI of 2 (HCV) or 1 (poliovirus and EV71), and the culture supernatant was collected 4 days (HCV) or 1 day (poliovirus and EV71) after infection. For WNV_KUN_, CHIKV, HSV-1, and HSV-2 infections, V5-RyDEN or V5-DHFR-expressing HeLa cells were infected at an MOI of 1 (for WNV_KUN_ and CHIKV) or an MOI of 0.1 (for HSV-1 and HSV-2), and the culture supernatant was collected 48 h (for KUNV) or 72 h (for CHIKV, HSV-1, and HSV-2) after infection. HIV-1 infection of V5-RyDEN or V5-DHFR-expressing Jurkat cells were carried out by exposing the virus (MOI of 0.005) for 2 h, and the level of virus replication was measured with a p24^Capsid^ concentration in a culture supernatant of infected cells [35]. For HAdV-3 infection, V5-RyDEN or V5-RLuc-expressing A549 cells were infected with a virus at an MOI of 1, and the culture supernatant was collected at 24 h.

### Virus entry assay

Virus entry assay was performed as reported by Le Sommer *et al*. [40]. Huh7.5 cells stably expressing V5-RyDEN or V5-RLuc, which had been seeded in a 24-well plate at a density of 5 x 10^4^/well 1 day before infection, were incubated with DENV-2 at an MOI of 5 at 37°C for 2 h. Uninternalized virus particles were removed by washing the cells twice with cold PBS, followed by a 3-min exposure to 1 M NaCl and 50 mM Na_2_CO_3_, pH 9.5. After washing with cold PBS three more times, total RNA was extracted and cell-associated DENV RNA was analyzed by qRT-PCR analysis.

### Viral RNA transfection assay

DENV-2 RNA was first extracted from the virus supernatant using QIAamp Viral RNA Mini Kit (Qiagen). For transfecting the isolated viral RNA, Huh7.5 cells that expressed V5-RyDEN or V5-RLuc were preseeded in a 6-well plate at a density of 5 × 10^5^/well 1 day before transfection and transfected with DENV-2 RNA equivalent to 6.7 × 10^7^ PFU using Lipofectamine 2000 (Life Technologies). After 3 days, the culture supernatant was collected to measure the infectious titer of extracellular virus via plaque assay.

### Cell-based reporter assay

A stable A549 cell line expressing a self-replicating DENV replicon was generated by the transfection of *in vitro* transcribed and 5'-capped genomic RNA of the DENV-2 NGC strain, in which structural genes had been replaced with puromycin-resistant gene and *Renilla* luciferase gene (DENrepPAC2A-Rluc), and subsequent selection with 5 μg/ml of puromycin as described previously [42]. Established cells were seeded in a 24-well plate at a density of 2 x 10^4^ cells/well and, on the next day, transfected with 4–400 ng of V5-RyDEN or V5-BAP-expressing pcDNA3.1 by Lipofectamine 2000. Forty-eight hours after transfection, cells were harvested and subjected to a luciferase assay using the Renilla Luciferase Glow Assay Kit (Thermo Scientific), as described previously [70]. As an inhibition control experiment, RLuc replicon-expressing A549 cells were also transfected with 10 nM siRNA duplex against DENV NS3 [42] or a scrambled siRNA duplex using siLentFect (Bio-Rad) and analyzed by luciferase assay.

To construct a mutant DENV reporter construct, DENrepPAC2A-Rluc GVD, aspartic acid (D) at position 663 of NS5 was changed to valine (V) [58,73] by QuikChange II XL Site-Directed Mutagenesis Kit (Agilent) using DENrepPAC2A-Rluc as a template plasmid DNA. RNA of DENrepPAC2A-Rluc WT and GVD were *in vitro* transcribed from *Xba*I-digested plasmid DNA using MEGAscript T7 Transcription Kit (Life Technologies) in the presence of m7GpppA cap analogue (NEB) and purified by RNeasy Mini Kit (Qiagen). For reporter assay, V5-RyDEN and V5-DHFR-expressing HepG2 cells, which had been preseeded in 24-well plates at 1 x 10^5^ cells/well density, were transfected with 500 ng of transcribed RNA using Lipofectamine 2000, and 4 and 8 h after transfection, the cells were subjected to luciferase activity assay.

### ISG expression analysis

V5-RyDEN or V5-BAP-expressing plasmid DNA was constructed using pcDNA3.1/nV5-DEST (Life Technologies) by a Gateway BP reaction. To construct the expression plasmid of STING, ORF of STING, which was fused with the N-terminal HA tag sequence, was generated by RT-PCR using mRNA from HeLa cells and cloned into the *EcoR*V site of pcDNA3.1 (Life Technologies). Constructed plasmid DNA (500 ng) was transfected to HepG2 cells (preseeded in a 24-well plate at 5 x 10^4^ cells/well density 1 day before transfection) using jetPRIME (Polyplus Transfection) and incubated for 48 h. Total RNA was extracted using the RNeasy Mini Kit (Qiagen) and was subjected to RT-qPCR analysis using SsoAdvanced SYBR Green Supermix and primers listed in S1 Table.

### Affinity purification and mass spectrometry analysis

An ORF of RyDEN or BAP was cloned into a lentiviral vector, pYK005C-NTAP-Bla in which a TAP tag consisting of two IgG binding units, a tobacco etch virus (TEV) protease cleavage site, and a streptavidin-binding peptide [44] had been added upstream of the Gateway unit in pYK005C-Bla by LR reaction. A VSV-G-pseudotyped lentiviral vector produced using 293T cells was used to transduce HepG2 cells. The transduced cells were selected in the presence of 10 μg/ml of blasticidin. Expression of N-terminal TAP-tagged RyDEN and BAP proteins were confirmed by immunoblotting using nonspecific rabbit IgG (primary antibody) and HRP-conjugated anti-rabbit IgG as a secondary antibody.

For affinity purification analysis, TAP-fused protein-expressing cells (90% confluence in a 100-mm culture dish) were harvested from a total of 12 dishes, washed twice in PBS that contained 10 mM EDTA, and lysed in 7.8 ml of TAP lysis buffer (50 mM Tris-HCl, pH8.0, 0.5 mM EDTA, 1 mM DTT, 150 mM NaCl, 0.2% NP-40, protease inhibitors) on ice for 40 min. Cell debris was removed by centrifugation for 10 min at 10,000 × *g*. The supernatants were incubated with 840 μl of IgG Sepharose 6 Fast Flow (50% slurry, GE Healthcare) at 4°C for 2 h. Beads were washed three times with TAP washing buffer (50 mM Tris-HCl, pH 8.0, 0.5 mM EDTA, 1 mM DTT, 300 mM NaCl, 1% NP-40). Proteins were eluted in 1.24 ml of TAP elution buffer (50 mM Tris-HCl, pH 8.0, 0.5 mM EDTA, 1 mM DTT, 150 mM NaCl, 0.2% NP-40) containing 60 U of TEV protease (Life Technologies) at 4°C overnight. The eluted protein sample was concentrated using trichloroacetic acid and separated by a 10% SDS-PAGE gel. Mass spectrometric identification of proteins was performed using MALDI TOF-TOF MS at Protein and Proteomics Centre, Department of Biological Sciences, National University of Singapore.

### Co-immunoprecipitation analysis

To construct deletion mutants of RyDEN, cDNA covering amino acid positions 51–291, 101–291, 151–291, and 1–250 were amplified by PCR. Site-directed mutagenesis of RyDEN for substitutions of arginine and lysine to alanine in NLS-L (amino acid positions 121–137) was performed by the overlapping PCR technique using two complementary primers flanking both ends of the RyDEN ORF and two internal mutagenic 25-nucleotide primers. After the first round of PCR, the two mutated DNA fragments (5’ and 3’ parts) were annealed, and a second round of PCR was carried out using the complementary primers. All PCR fragments were gel purified, cloned into pDONR221, and, after confirmation of sequences, transferred to the Gateway unit of pYK005C-Bla. The VSV-G-pseudotyped lentiviral vectors were produced using HEK293T cells and were used to transduce Huh7.5 (for the deletion mutant experiment) or HepG2 (for the NLS-L mutant experiment), followed by selection with blasticidin (10 μg/ml).

Stable cell lines (90% confluence in a 100-mm culture dish) that expressed V5-tagged RyDEN (WT, deletion mutants, and NLS-L mutants), or control RLuc were lysed using 1.1 ml of TAP lysis buffer on ice for 40 min and cleared by centrifugation. Five-hundred microliters of cell lysate were then incubated with 3 μl of an anti-PABPC1 mouse monoclonal antibody (10E10, Santa Cruz Biotechnology) at 4°C for 2 h with rotation, followed by the addition of 30 μl of Protein A/G agarose beads (Santa Cruz Biotechnology) and another 2 h of incubation at 4°C. The bound complexes were washed five times with TAP elution buffer and eluted in SDS sample buffer for immunoblotting analysis. In the co-immunoprecipitation experiments, V5-RLuc was used as a control protein to avoid overlapping with IgG light chain of the anti-PABPC1 antibody (used for pull-down) on immunoblots.

### siRNA-based gene knockdown experiment

siRNA duplexes that target human PABPC1 (siPABPC1: 5’-AGG CGA UGC UCU ACG AGA AdTdT-3’) and human LARP1 (siLARP1: 5’-GAA UGG AGA UGA GGA UUG CdTdT-3’) and a negative control siRNA duplex (siCtrl) were purchased from SABio (Singapore). HepG2 cells preseeded in a 24-well plate at a density of 1 x 10^5^ cells/well 1 day before transfection were transfected with 50 nM siRNA duplex using jetPRIME and then inoculated with DENV-2 at an MOI of 1 48 h after transfection. Forty-eight hours after infection, the culture supernatant was collected and subjected to plaque assay to determine the viral infectious titer. At the same time, total RNA was extracted from infected cells and used for qRT-PCR to analyze the knockdown efficiency of PABPC1 and LARP1 mRNA using the primers listed in S1 Table.

### RIP assay

HepG2 cells that expressed V5- RyDEN (WT and NLS mutants) or V5-DHFR were seeded in a 6-well plate at a density of 5 x 10^5^ cells/well 1 day before infection and exposed to 2.5 x 10^6^ PFU of DENV-2 for 6 h. Cells were then washed with cold PBS three times and lysed with 300 μl of TAP lysis buffer on ice. After centrifugation at 10,000 x *g* for 10 min, the supernatant was incubated with 3 μl of an anti-V5 mouse monoclonal antibody in the presence of 100 ng/ml of tRNA (Sigma) at 4°C for 2 h with rotation, followed by the addition of 30 μl of protein A/G agarose beads (50% slurry in PBS, Pierce) and another 2 h of incubation at 4°C. The immune complex was washed with 500 μl of TAP washing buffer 5 times and suspended with RNase-free PBS. One-fourth of the suspension was used for immunoblotting to detect V5-tagged proteins, and the rest was used for RNA analysis. DENV RNA was extracted from the suspension using TRIzol (Life Technologies) and subjected to qRT-PCR using DENV 3’UTR-specific primers and a fluorescent probe (S1 Table).

### AlphaScreen-based RNA-binding assay

3’UTR sequence of DENV-2 NGC (nucleotide positions 10,271–10,724) was cloned into pEU vector containing SP6 promoter sequence (CellFree Sciences, Japan). A DNA fragment covering the upstream SP6 promoter and the downstream 3’UTR sequences (or DHFR sequence for nonspecific control RNA) was amplified from the pEU-based construct by PCR, which was then used for *in vitro* transcription in 25 μl of reaction containing 10 mM NTP, 0.25 mM biotinylated UTP (Roche Diagnostics), and 0.8 units/μl SP6 polymerase (CellFree Sciences). Resulting transcripts were column purified, followed by ethanol precipitation to remove free biotinylated UTP.

For production of recombinant proteins, a DNA fragment containing 5’ SP6 promoter, N-terminal tag (consisting of GST and FLAG units, separated by TEV protease cleavage site [GST-TEV-FLAG] for FLAG-tagged proteins [RyDEN WT, RyDEN NLS-L mutant, and DHFR], or GST unit and TEV protease site [GST-TEV] for GST-tagged proteins [PABPC1 and DHFR]), and the protein ORF sequences was amplified from plasmid DNA encoding RyDEN (WT or NLS-L mutant), DHFR, or PABPC1 by previously described split-primer PCR method [74] and used as a template for *in vitro* transcription. *In vitro* RNA transcription and subsequent translation of proteins using wheat germ cell-free protein production system were performed in 96-well plate by the bilayer diffusion method using ENDEXT technology (CellFree Sciences) according to the manufacturer’s protocol. The synthesized proteins were captured with glutathione Sepharose 4B (GE healthcare), and the beads were washed with PBS. Proteins were then eluted from beads using elution buffer (50 mM Tris-HCl, pH8.0, 100 mM NaCl) containing 0.4 U/μl TEV protease (for FLAG-tagged proteins) or 10 mM reduced glutathione (for GST-tagged proteins).

*In vitro* RNA binding assay was performed with 384-well OptiPlate by AlphaScreen technology (PerkinElmer). Twenty nanomolar of FLAG-tagged proteins were mixed with 20 nM of GST-tagged proteins and 3.5 ng/μl biotinylated (or non-biotinylated) DENV 3’UTR RNA (or control RNA) in 15 μl of the binding mixture containing reaction buffer (100 mM Tris-HCl, pH7.5, 100 mM NaCl, 1 mg/ml BSA, 0.01% Tween 20) at 16°C. After 1 h incubation, 10 μl of the detection mixture containing 0.2 μg/ml anti-FLAG mouse monoclonal antibody (Wako), 0.1 μl of streptavidin-coated donor beads and 0.1 μl of protein A-conjugated acceptor beads (PerkinElmer) in reaction buffer was added to the binding mixture, followed by incubation at 16°C for 1 h. Luminescent signal was analyzed by an EnVision microplate luminometer (PerkinElmer) [56].

### Puromycin labeling

V5-RyDEN and V5-DHFR-expressing HepG2 cells preseeded in a 12-well plate at a density of 2 x 10^5^ cells/well 1 day before assay were cultured with 10 or 20 μg/ml cycloheximide for 1 h. After the medium was changed, cells were further cultured in the presence of 10 μg/ml of puromycin (Clontech). Cells were harvested 40 min after puromycin pulse, and the cell lysate was subjected to immunoblotting using anti-puromycin mouse monoclonal antibody (3RH11, KeraFAST).

### Statistical analysis

All data are obtained by a representative set of at least three independent experiments, and the average values are shown with error bars indicating the standard deviation (SD). Statistical significance was performed using JMP Pro software version 11 (SAS Institute). *P* values below 0.05 (*P*<0.05, *; *P*<0.01, **; *P*<0.001, ***) were considered significant.

### Accession numbers

In this study, the following reference sequences were used to design oligonucleotides: DENV-2 NGC (AF038403.1); C19orf66 (NM_018381); PABPC1 (NM_002568.3); LARP1 (NM_015315.4); BAP (M13345.1); DHFR (J01609.1); ISG54 (NM_001547.4); ISG15 (NM_005101.3); LY6E (NM_002346.2); RIG-I (AF038963.1); IFN-β (M25460.1); GAPDH (NM_002046.5).

## Supporting Information

S1 Fig Massive cytopathic effect in Huh7.5 cells by DENV infection. Huh7.5 cells were infected with DENV-2 at MOIs of 0.1, 1, and 10. At 1, 3, and 7 days post infection, DENV-induced cell death was observed under light microscopy (A) and also analyzed by annexin V staining and flow cytometric analysis (B).
(TIF)

S2 Fig Inhibition of DENV replication by overexpressing RyDEN in HEK293T cells. HEK293T cells expressing V5-tagged RyDEN (gray) and DHFR (white) were established by lentiviral vector transduction and subsequent blasticidin selection. Cells were then infected with DENV-2 at an MOI of 1, and 2 days after infection, infectious titers in culture supernatants were analyzed by plaque assay. Statistical significance was determined by Student’s *t* test.
(TIF)

S3 Fig Enhancement of DENV infection by knockdown of RyDEN in human hepatoma cell lines. HepG2 and Huh7.5 cells. HepG2 (A and C) and Huh7.5 (B and D) cells stably expressing shRNA against RyDEN mRNA (sh1425 or sh3151) were created by lentiviral vector transduction and subsequent puromycin selection. (A and B) The expression level of RyDEN mRNA in RyDEN shRNA and control shRNA (shCtrl)-expressing cells were analyzed by qRT-PCR analysis and normalized to GAPDH mRNA levels. (C and D) shRNA-expressing HepG2 (C) and Huh7.5 (D) cells were infected with DENV-2 at an MOI of 1, and 2 days after infection, viral titer in culture supernatant was measured my plaque assay. Statistical significance was determined by one-way ANOVA with Dunnett’s multiple comparison test.
(TIF)

S4 Fig Induction of RyDEN expression in various human cell lines by type I IFN. HeLa (cervical carcinoma), HepG2 (hepatoma), Huh7.5 (hepatocellular carcinoma), HEK293T (embryonic kidney), A549 (lung adenocarcinoma), Jurkat (lymphoblastoid T), and THP-1 (monocytic leukemia, ATCC TIB-202) cells were cultured in the presence (gray bars) or absence (white bars) of IFN-α/ω (1,000 U/ml). Total RNA was isolated 24 h after treatment and subjected to qRT-PCR analysis to detect RyDEN mRNA. The levels of RyDEN expression were normalized to GAPDH mRNA levels and expressed as relative to untreated HeLa cells (dashed line). In a parallel experiment, HepG2 cells were infected with DENV-2 at an MOI of 5, and then, total RNA isolated 24 h after infection was subjected to qRT-PCR analysis (black bar).
(TIF)

S5 Fig Comparison of inhibitory effects of RyDEN and other antiviral agents on the activity of DENV subgenomic RNA replicon. A549 cells harboring the DENV-2 RNA replicon carrying the luciferase reporter gene (in 24-well plate at 5 x 104 cells/well 1 day before assay) were transfected with 400 ng of V5-protein-expressing plasmid DNA (V5-RyDEN or V5-BAP) or treated with 1,000 U/ml of IFN-α/ω, 10 μg/ml of mycophenolic acid (MPA), or 0.02% DMSO (control for MPA). Forty-eight hours after transfection/treatment, cells were harvested and subjected to luciferase assay. Luciferase activity in the cell lysate was normalized to total protein concentration. Statistical significance was determined by one-way ANOVA with Dunnett’s multiple comparison test.
(TIF)

S6 Fig MS/MS spectra of protein fragments isolated by affinity purification. Protein complex isolated with TAP-RyDEN by affinity purification was separated by SDS-PAGE. Bands of interest (around 150 kDa [identified as LARP1, top panel], > 70 kDa [identified as PABPC1, middle panel], and >40 kDa [identified as RyDEN, bottom panel] bands) were cut from gel and digested with trypsin. The resulting peptides were subjected to tandem mass spectrum analysis and detected ions were analyzed using the Mascot search engine (Matrix Science). Amino acid sequences matched are shown in red.
(TIF)

S7 Fig Immunoblotting analysis of siPABPC1-transfected cells. HepG2 cells were transfected with 50 nM siRNA duplex against PABPC1 (siPABPC1) and negative control siRNA duplex (siCtrl) and 48 h after transfection, cells were subjected to immunoblotting analysis using anti-PABPC1 antibody (top panel). Bottom panel, immunoblotting analysis to detect actin. Molecular weight standards are indicated at the left.
(TIF)

S8 Fig Distribution of RyDEN in various human cell lines. (A) HepG2, Huh7.5, HeLa, HEK293T, and A549 cells were treated with 1,000 units/ml of IFN-α/ω for 24 h and subjected to IFA using anti-RyDEN rabbit serum and FITC-conjugated anti-rabbit secondary antibody (top row). (B) V5- RyDEN-expressing HepG2 cells were either treated with 1,000 units/ml of IFN-α/ω or infected with DENV-2 at MOI of 10, and 48 h after treatment/infection, subjected to IFA using anti-V5 antibody and Alexa Fluor 488-conjugated anti-mouse secondary antibody (top row). Cell nuclei were stained with DAPI (center rows). Merged images are shown in the bottom rows.
(TIF)

S9 Fig Purification of recombinant RyDEN and PABPC1 proteins. All proteins used in AlphaScreen-based *in vitro* RNA-binding assay were produced by the wheat germ cell-free system and affinity purified using glutathione Sepharose beads. As for FLAG-tagged proteins, N-terminal GST-tag was removed by TEV protease cleavage. Purified proteins were resolved by SDS-PAGE and visualized by CBB staining. MW, molecular weight standard. BSA was used to determine protein concentration.
(TIF)

S10 Fig Competition assay of RyDEN, PABPC1, and DENV 3'UTR interaction. AlphaScreen-based *in vitro* RNA binding assay using 20 nM FLAG-RyDEN, 20 nM GST-PABPC1, and 3.5 ng/ml biotinylated DENV 3'UTR RNA was performed in the presence of unlabeled 3'UTR RNA (3.5–87.5 ng/μl). Statistical significance was determined by one-way ANOVA with Dunnett’s multiple comparison test.
(TIF)

S11 Fig Effect of RyDEN expression on Sindbis virus (SINV) replication. HeLa cells expressing V5-RyDEN and DHFR were infected with SINV at an MOI of 1, and 24 h after infection, infectious titers in culture supernatants were analyzed by plaque assay.
(TIF)

S1 Table Oligonucleotide primers and fluorescent probes used in qRT-PCR. (TIF)
