# Supplementary figures and images for: Characterization of RyDEN (C19orf66) as an Interferon-Stimulated Cellular Inhibitor against Dengue Virus Replication
Source: PLoS Pathog. 2016 Jan 6;12(1):e1005357. doi: 10.1371/journal.ppat.1005357 (PMC4703206; doi:10.1371/journal.ppat.1005357)

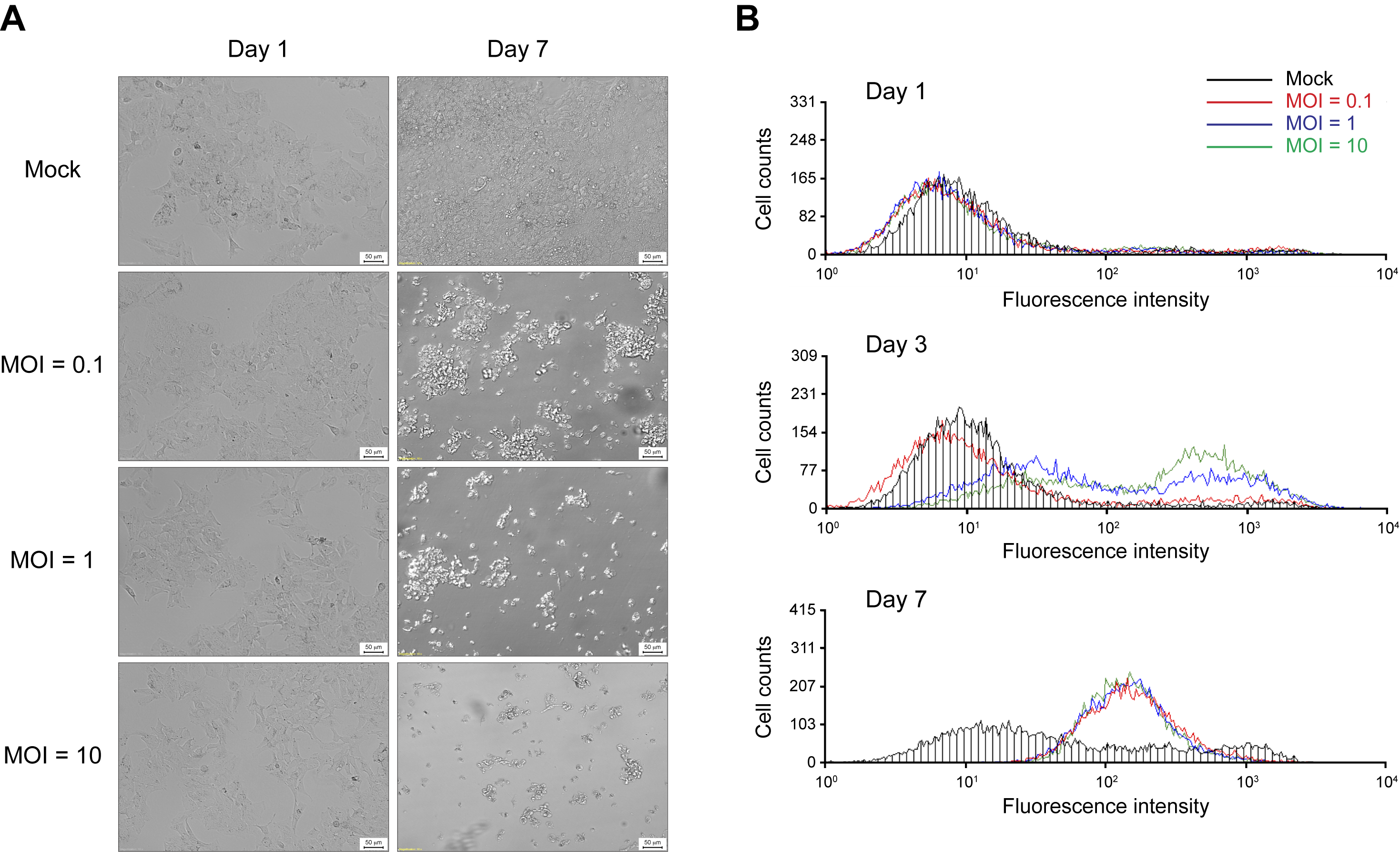

Supplement: S1 Fig — Huh7.5 cells were infected with DENV-2 at MOIs of 0.1, 1, and 10. At 1, 3, and 7 days post infection, DENV-induced cell death was observed under light microscopy (A) and also analyzed by annexin V staining and flow cytometric analysis (B). (TIF) [file ppat.1005357.s001.tif]

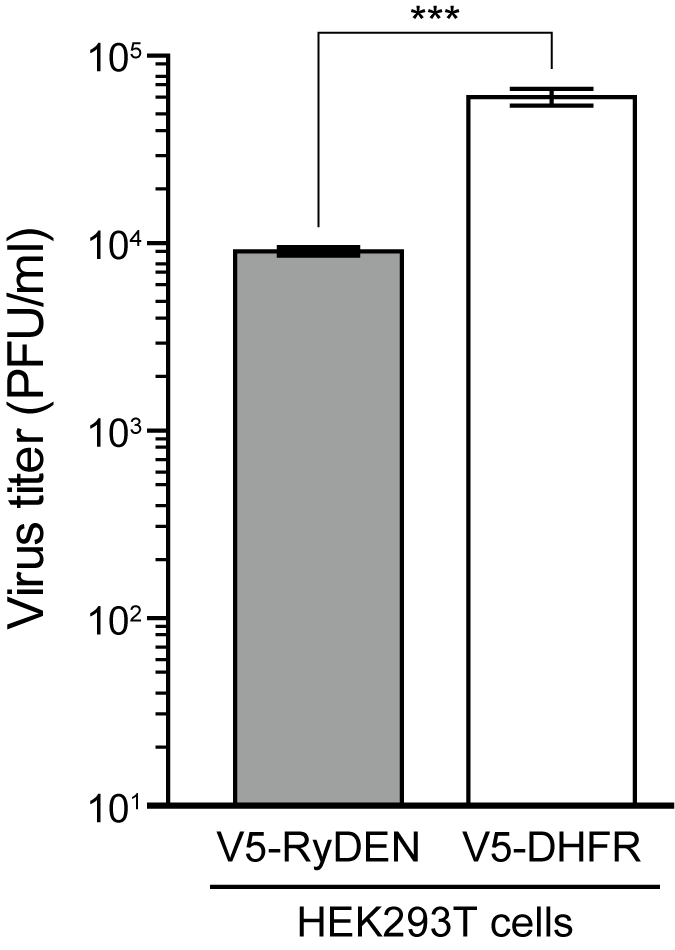

Supplement: S2 Fig — HEK293T cells expressing V5-tagged RyDEN (gray) and DHFR (white) were established by lentiviral vector transduction and subsequent blasticidin selection. Cells were then infected with DENV-2 at an MOI of 1, and 2 days after infection, infectious titers in culture supernatants were analyzed by plaque assay. Statistical significance was determined by Student’s t test. (TIF) [file ppat.1005357.s002.tif]

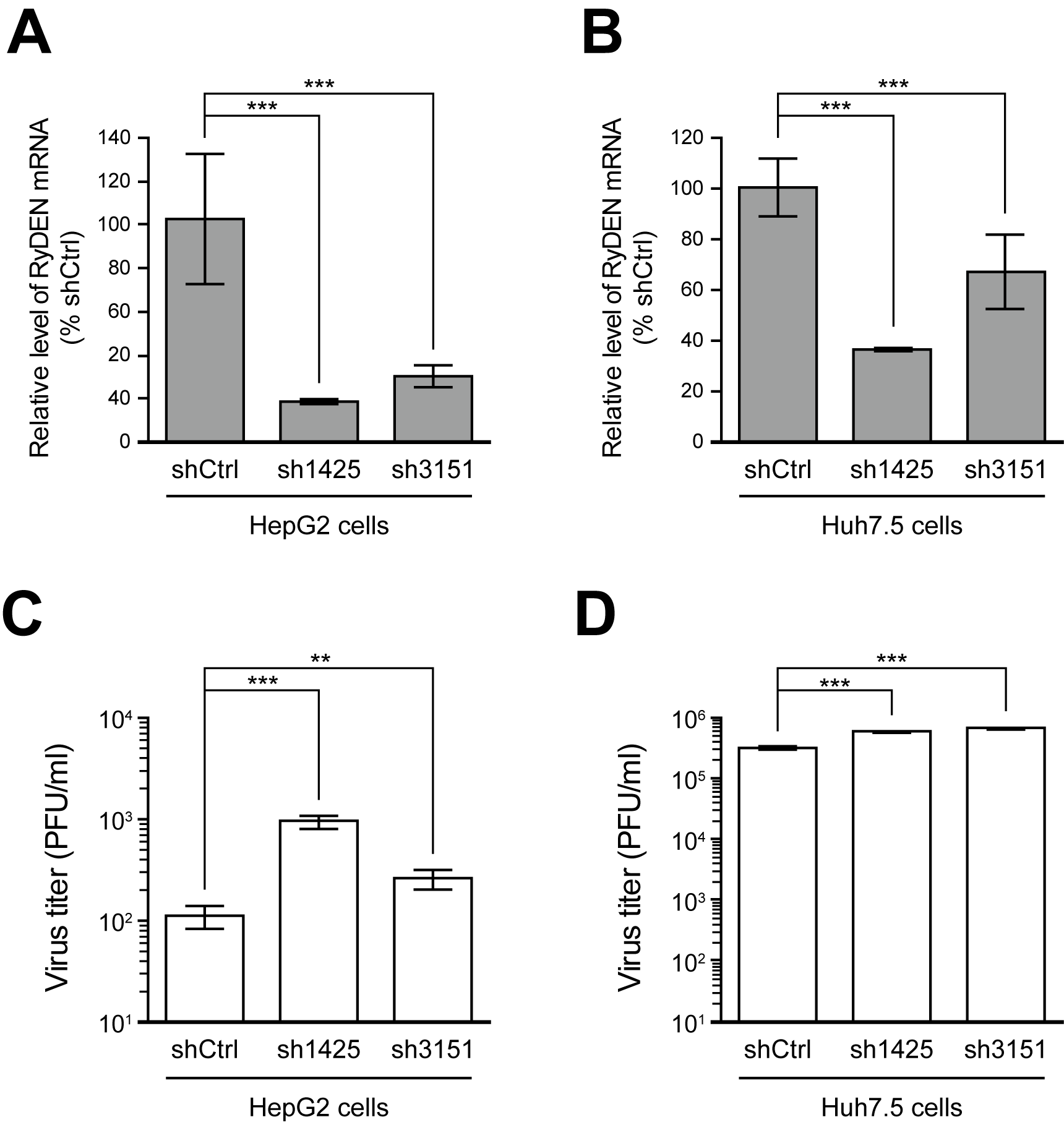

Supplement: S3 Fig — HepG2 and Huh7.5 cells. HepG2 (A and C) and Huh7.5 (B and D) cells stably expressing shRNA against RyDEN mRNA (sh1425 or sh3151) were created by lentiviral vector transduction and subsequent puromycin selection. (A and B) The expression level of RyDEN mRNA in RyDEN shRNA and control shRNA (shCtrl)-expressing cells were analyzed by qRT-PCR analysis and normalized to GAPDH mRNA levels. (C and D) shRNA-expressing HepG2 (C) and Huh7.5 (D) cells were infected with DENV-2 at an MOI of 1, and 2 days after infection, viral titer in culture supernatant was measured my plaque assay. Statistical significance was determined by one-way ANOVA with Dunnett’s multiple comparison test. (TIF) [file ppat.1005357.s003.tif]

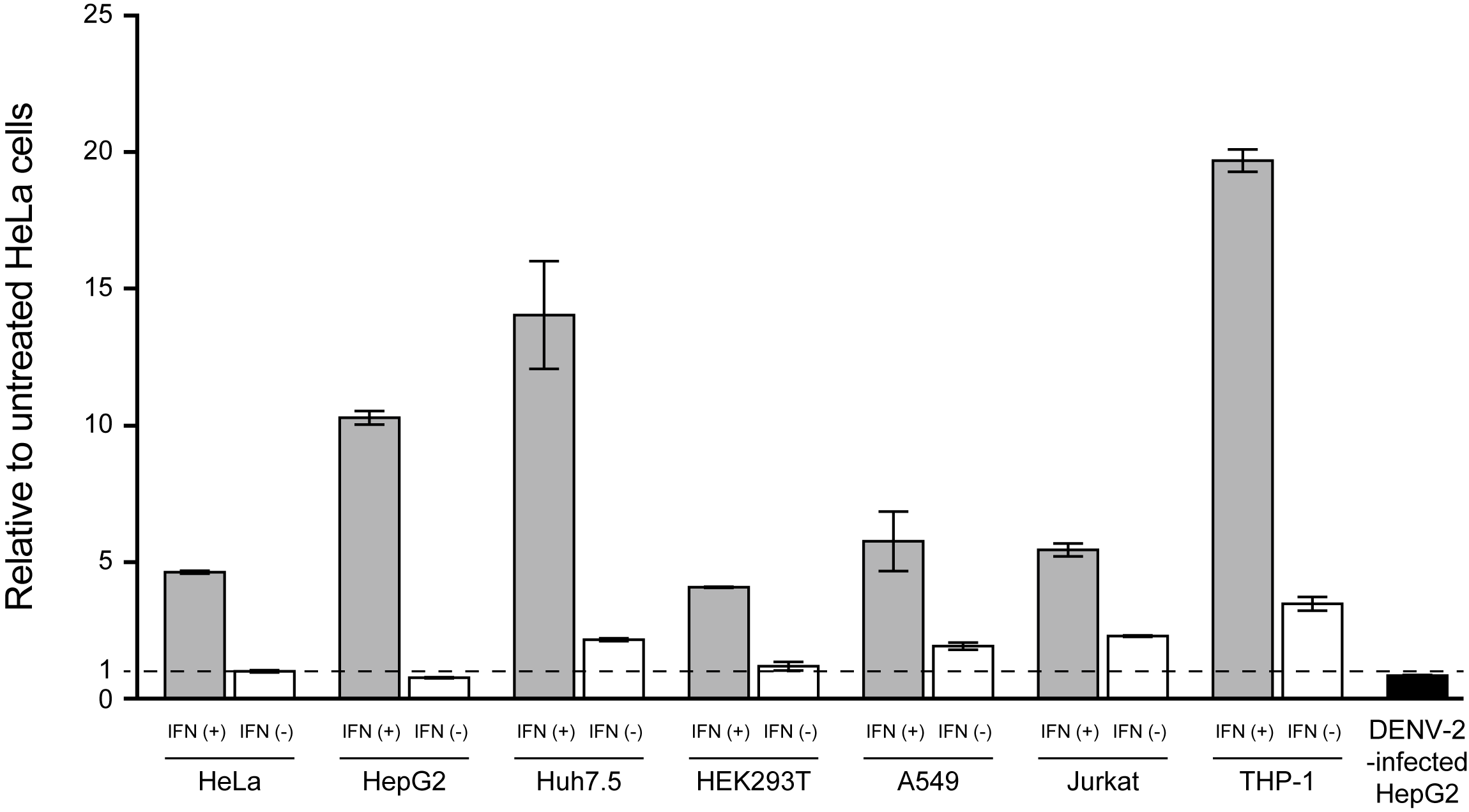

Supplement: S4 Fig — HeLa (cervical carcinoma), HepG2 (hepatoma), Huh7.5 (hepatocellular carcinoma), HEK293T (embryonic kidney), A549 (lung adenocarcinoma), Jurkat (lymphoblastoid T), and THP-1 (monocytic leukemia, ATCC TIB-202) cells were cultured in the presence (gray bars) or absence (white bars) of IFN-α/ω (1,000 U/ml). Total RNA was isolated 24 h after treatment and subjected to qRT-PCR analysis to detect RyDEN mRNA. The levels of RyDEN expression were normalized to GAPDH mRNA levels and expressed as relative to untreated HeLa cells (dashed line). In a parallel experiment, HepG2 cells were infected with DENV-2 at an MOI of 5, and then, total RNA isolated 24 h after infection was subjected to qRT-PCR analysis (black bar). (TIF) [file ppat.1005357.s004.tif]

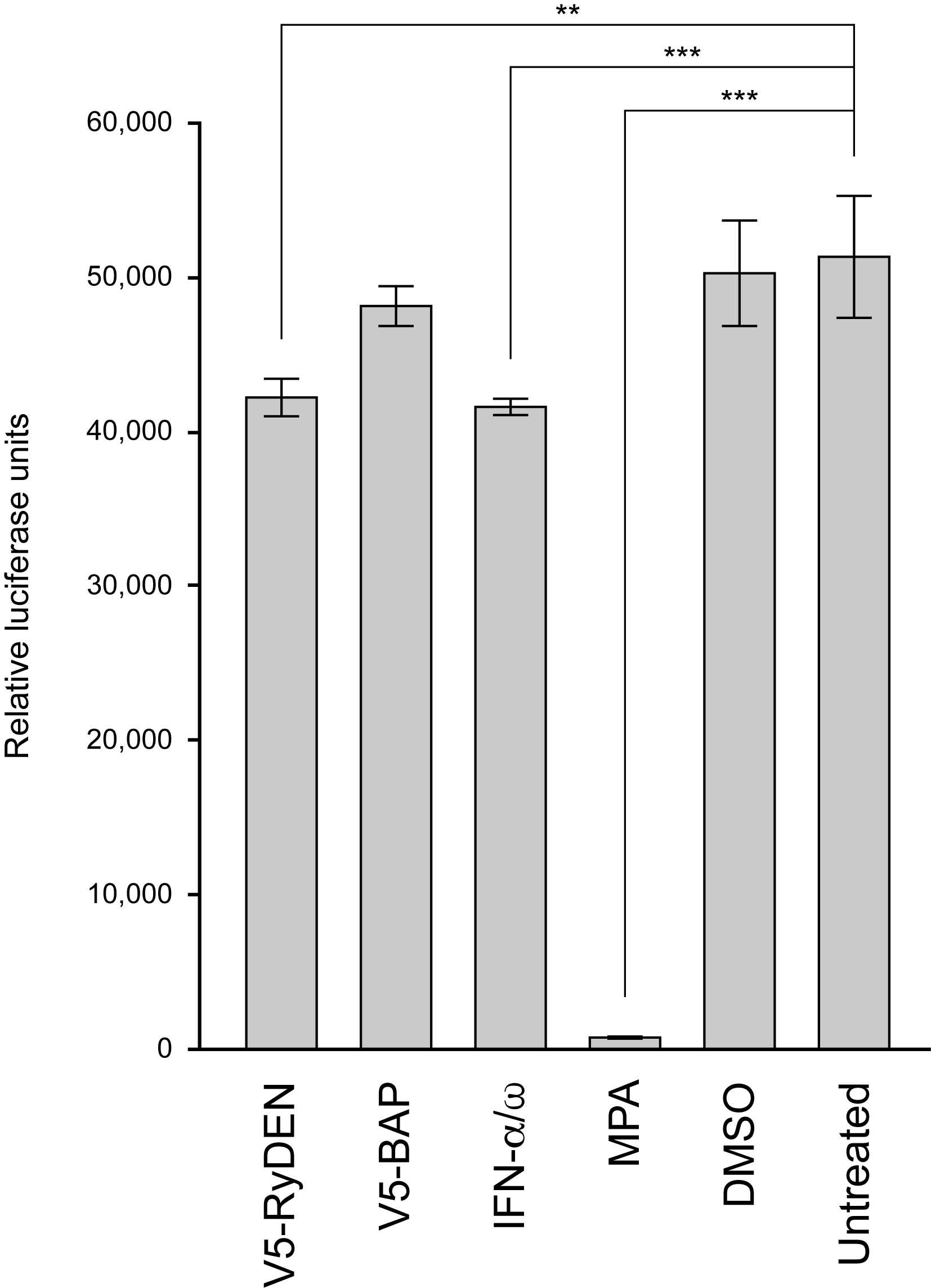

Supplement: S5 Fig — A549 cells harboring the DENV-2 RNA replicon carrying the luciferase reporter gene (in 24-well plate at 5 x 10^4^ cells/well 1 day before assay) were transfected with 400 ng of V5-protein-expressing plasmid DNA (V5-RyDEN or V5-BAP) or treated with 1,000 U/ml of IFN-α/ω, 10 μg/ml of mycophenolic acid (MPA), or 0.02% DMSO (control for MPA). Forty-eight hours after transfection/treatment, cells were harvested and subjected to luciferase assay. Luciferase activity in the cell lysate was normalized to total protein concentration. Statistical significance was determined by one-way ANOVA with Dunnett’s multiple comparison test. (TIF) [file ppat.1005357.s005.tif]

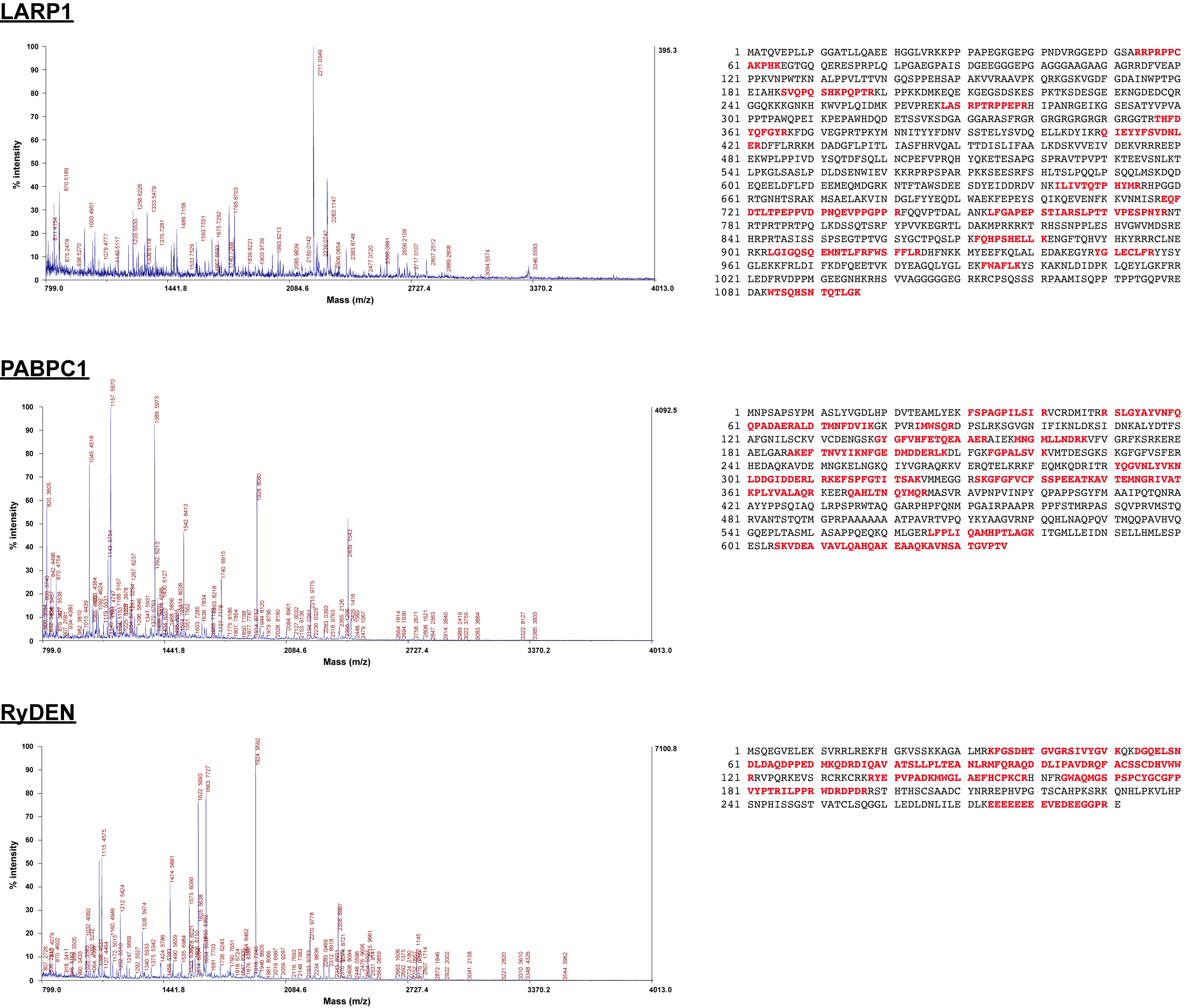

Supplement: S6 Fig — Protein complex isolated with TAP-RyDEN by affinity purification was separated by SDS-PAGE. Bands of interest (around 150 kDa [identified as LARP1, top panel], > 70 kDa [identified as PABPC1, middle panel], and >40 kDa [identified as RyDEN, bottom panel] bands) were cut from gel and digested with trypsin. The resulting peptides were subjected to tandem mass spectrum analysis and detected ions were analyzed using the Mascot search engine (Matrix Science). Amino acid sequences matched are shown in red. (TIF) [file ppat.1005357.s006.tif]

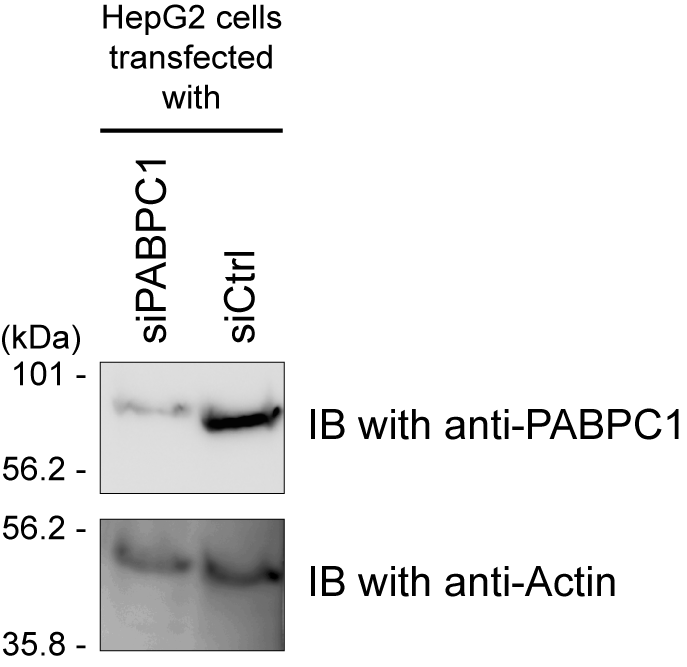

Supplement: S7 Fig — HepG2 cells were transfected with 50 nM siRNA duplex against PABPC1 (siPABPC1) and negative control siRNA duplex (siCtrl) and 48 h after transfection, cells were subjected to immunoblotting analysis using anti-PABPC1 antibody (top panel). Bottom panel, immunoblotting analysis to detect actin. Molecular weight standards are indicated at the left. (TIF) [file ppat.1005357.s007.tif]

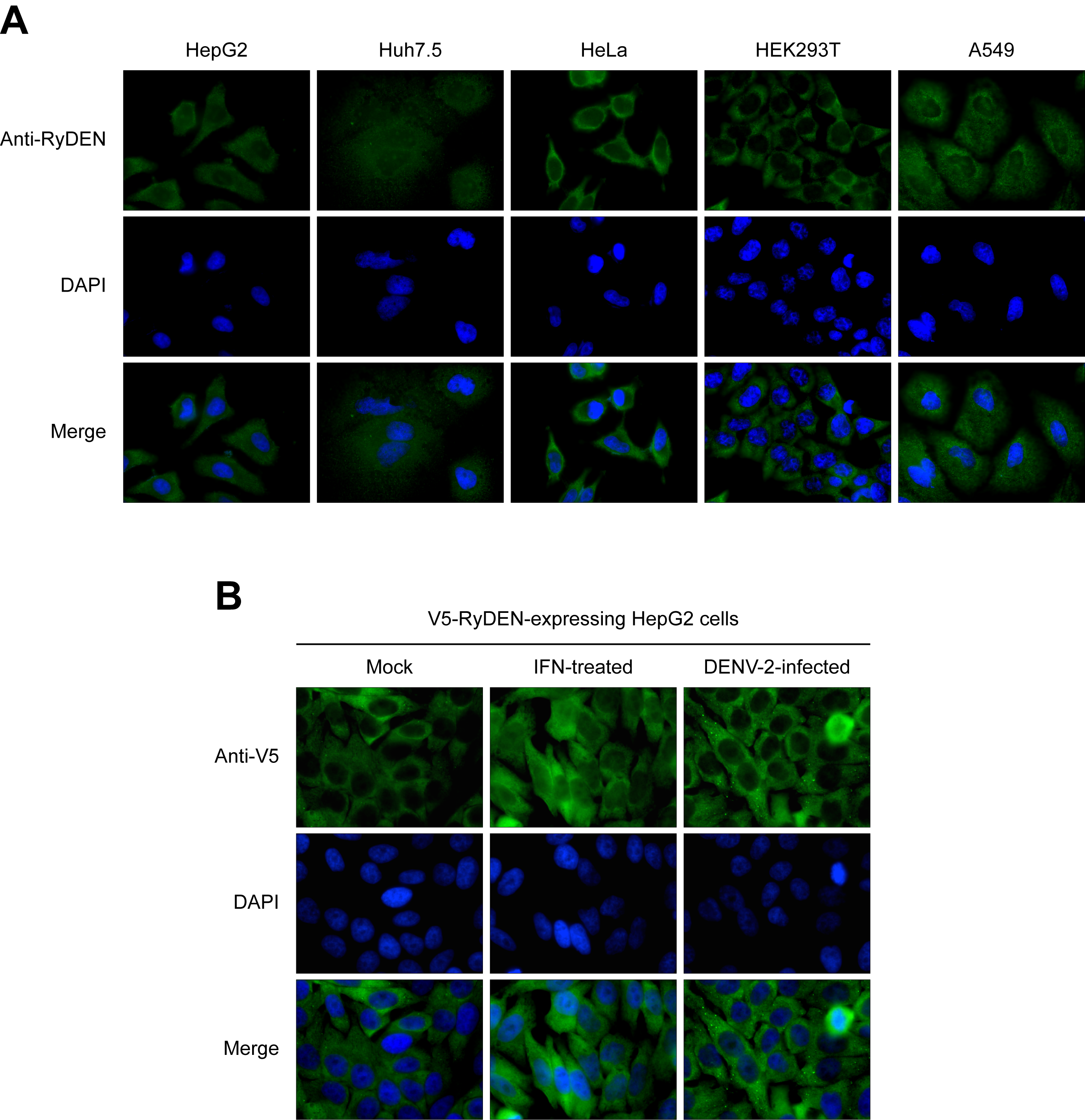

Supplement: S8 Fig — (A) HepG2, Huh7.5, HeLa, HEK293T, and A549 cells were treated with 1,000 units/ml of IFN-α/ω for 24 h and subjected to IFA using anti-RyDEN rabbit serum and FITC-conjugated anti-rabbit secondary antibody (top row). (B) V5- RyDEN-expressing HepG2 cells were either treated with 1,000 units/ml of IFN-α/ω or infected with DENV-2 at MOI of 10, and 48 h after treatment/infection, subjected to IFA using anti-V5 antibody and Alexa Fluor 488-conjugated anti-mouse secondary antibody (top row). Cell nuclei were stained with DAPI (center rows). Merged images are shown in the bottom rows. (TIF) [file ppat.1005357.s008.tif]

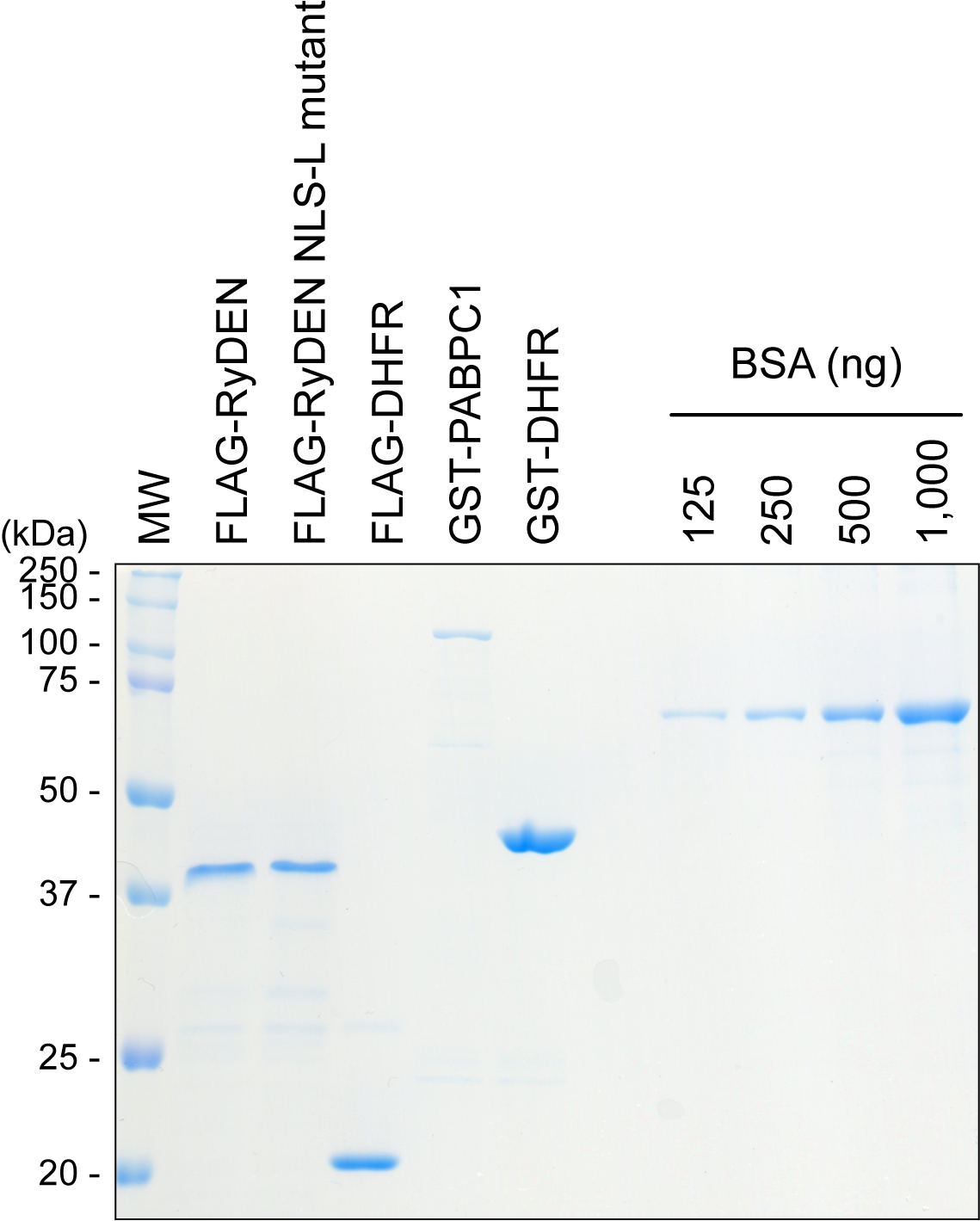

Supplement: S9 Fig — All proteins used in AlphaScreen-based in vitro RNA-binding assay were produced by the wheat germ cell-free system and affinity purified using glutathione Sepharose beads. As for FLAG-tagged proteins, N-terminal GST-tag was removed by TEV protease cleavage. Purified proteins were resolved by SDS-PAGE and visualized by CBB staining. MW, molecular weight standard. BSA was used to determine protein concentration. (TIF) [file ppat.1005357.s009.tif]

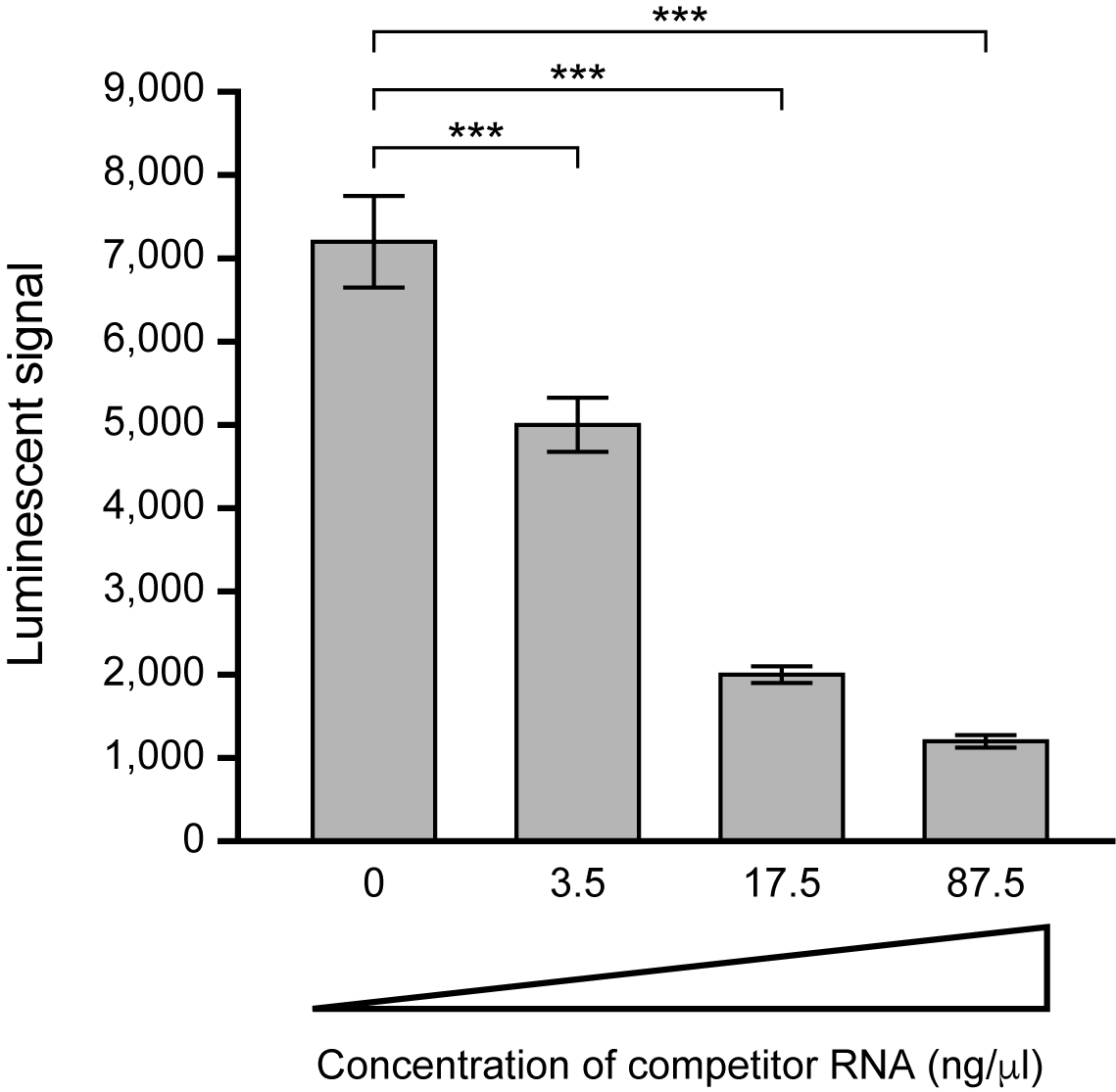

Supplement: S10 Fig — AlphaScreen-based in vitro RNA binding assay using 20 nM FLAG-RyDEN, 20 nM GST-PABPC1, and 3.5 ng/ml biotinylated DENV 3'UTR RNA was performed in the presence of unlabeled 3'UTR RNA (3.5–87.5 ng/μl). Statistical significance was determined by one-way ANOVA with Dunnett’s multiple comparison test. (TIF) [file ppat.1005357.s010.tif]

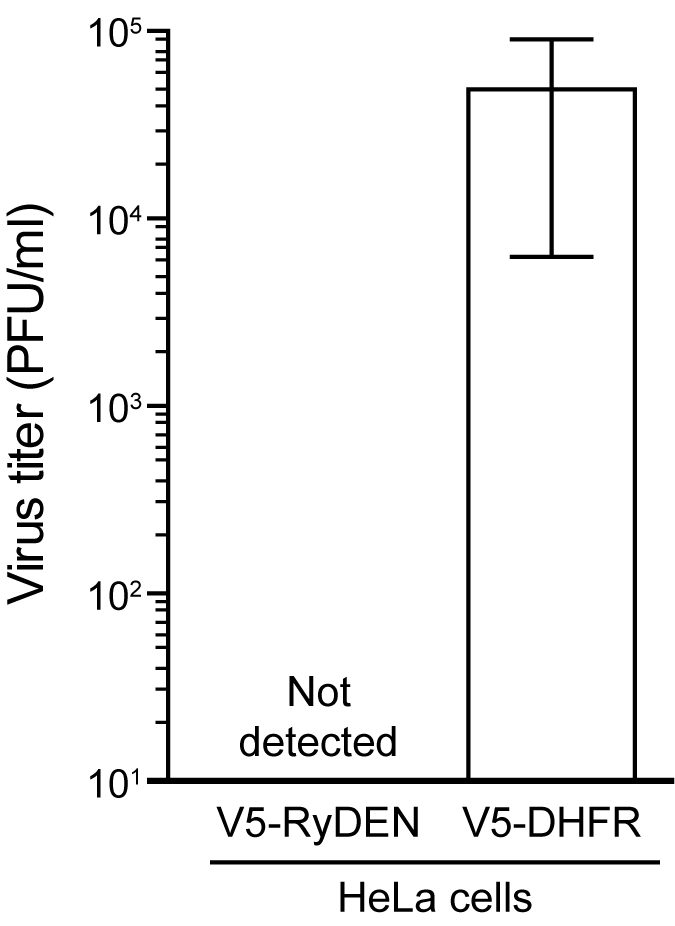

Supplement: S11 Fig — HeLa cells expressing V5-RyDEN and DHFR were infected with SINV at an MOI of 1, and 24 h after infection, infectious titers in culture supernatants were analyzed by plaque assay. (TIF) [file ppat.1005357.s011.tif]

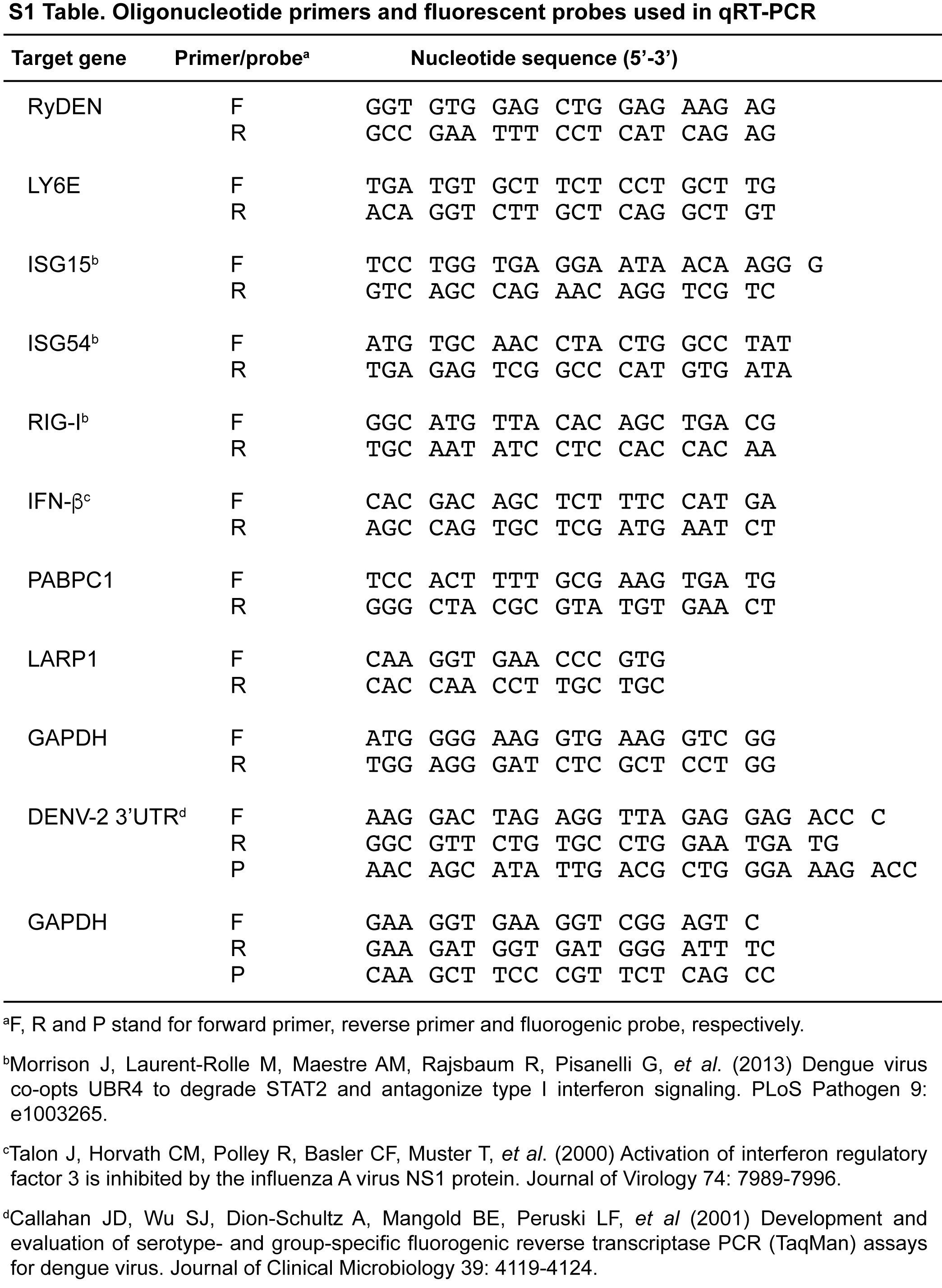

Supplement: S1 Table — (TIF) [file ppat.1005357.s012.tif]
